# Amniotic fluid stem cell-derived vesicles protect from VEGF-induced endothelial damage

**DOI:** 10.1038/s41598-017-17061-2

**Published:** 2017-12-04

**Authors:** S. Sedrakyan, V. Villani, S. Da Sacco, N. Tripuraneni, S. Porta, A. Achena, M. Lavarreda-Pearce, A. Petrosyan, H. Soloyan, R. E. De Filippo, B. Bussolati, L. Perin

**Affiliations:** 1 GOFARR Laboratory for Organ Regenerative Research and Cell Therapeutics in Urology, Children’s Hospital Los Angeles, Division of Urology, Saban Research Institute, University of Southern California, Los Angeles, California USA; 2 Department of Molecular Biotechnology and Health Sciences, University of Torino, Torino, Italy

## Abstract

Injection of amniotic fluid stem cells (AFSC) delays the course of progression of renal fibrosis in animals with Alport Syndrome, enhancing kidney function and improving survival. The mechanisms responsible for these protective outcomes are still largely unknown. Here, we showed that vascular endothelial growth factor (VEGF) signaling within the glomeruli of Alport mice is strongly elevated early on in the disease, causing glomerular endothelial cell damage. Intraventricular injected AFSC that homed within the glomeruli showed strong modulation of the VEGF activity, particularly in glomerular endothelial cells. To investigate this phenomenon we hypothesized that extracellular vesicles (EVs) produced by the AFSC could be responsible for the observed renoprotection. AFSC derived EVs presented exosomal and stem cell markers on their surface membrane, including VEGFR1 and VEGFR2. EVs were able to modulate VEGF in glomerular endothelial cells by effectively trapping the excess VEGF through VEGFR1-binding preventing cellular damage. In contrast, VEGFR1/sVEGFR1 knockout EVs failed to show similar protection, thus indicating that VEGF trapping is a potentially viable mechanism for AFSC-EV mediated renoprotection. Taken together, our findings establish that EVs secreted by AFSC could target a specific signaling pathway within the glomerulus, thus representing a new potential glomerulus-specific targeted intervention.

## Introduction

The complex local autocrine/paracrine signaling between podocytes and glomerular endothelial cells (GEC) is of critical importance for the homeostatic balance of the filtration barrier^1^. In particular, podocytes secrete various factors that act directly on the glomerular endothelium^2,3^. In recent years multiple studies have demonstrated that VEGF signaling plays a key role in the development and maintenance of glomerular capillary network and endothelial permeability^4,5^. An angiogenic imbalance between VEGF (specifically VEGF-A), VEGF receptor 2 (VEGFR2) and the soluble vascular endothelial growth factor receptor 1 (sVEGFR1, a truncated variant of the VEGF receptor 1, VEGFR1) has been reported in many diseases, including kidney disease where modulation of VEGF signaling correlates with impaired endothelial fenestrations, endothelial dysfunction and increased proteinuria^5–9^. Although the therapeutic use of compounds with anti-VEGF activity may prevent proteinuria in endothelial murine models of diabetic nephropathy^10,11^, the significance of VEGF/VEGFRs/sVEGFR1 modulation within the glomerular milieu, its contribution to GEC damage and progression of chronic kidney disease (CKD) is still not clearly understood.

We previously demonstrated that stem cells derived from amniotic fluid (AFSC) are renoprotective and significantly delayed disease progression in a mouse model of Alport Syndrome (AS, where a mutation in any of the collIVα3,α4,α5 genes results in the disruption of the glomerular basement membrane (GBM), podocyte effacement and renal failure) via preservation of podocyte number and maintenance of glomerular function^12^. The renoprotection by AFSC could possibly be ascribed to their ability to secrete various trophic mediators able to stimulate endogenous glomerular repair mechanisms. In this context, stem cell-derived extracellular vesicles (EVs), which are important cell-to-cell communication vehicles^13^, are suggested to be involved in tissue protective mechanisms^14,15^. At present, the mechanism(s) responsible for the therapeutic effect of AFSC on GEC damage and in particular their possible modulation of the VEGF pathway within the glomerulus has not yet been investigated.

In the present study, we found changes in VEGF signaling activity within the Alport glomeruli, particularly during the initiation phase of the disease. Injected AFSC that lodged within glomerular capillaries modulated VEGF/sVEGFR1 levels, thus preventing further endothelial damage, possibly by activating endogenous repair mechanisms. Specifically, we confirmed that AFSC release EVs that express various surface markers, including VEGFR1 and VEGFR2, and can modulate VEGF/VEGFRs signaling in damaged GEC by decreasing the bio-availability of excess VEGF. In conclusion, our data confirm the ability of AFSC to ameliorate renal damage and establish that their secreted EVs could target a specific signaling pathway re-establishing GEC function, thus representing a potentially new glomerulus-specific targeted intervention.

## Results

### VEGF/VEGFRs/sVEGFR1 signaling characterization within Alport glomerulus

To investigate the role of VEGF in AS progression, we determined if VEGF signaling is altered within the glomeruli of Alport mice. The specific VEGF isoform we studied is the VEGF-A.

**Figure 1:**
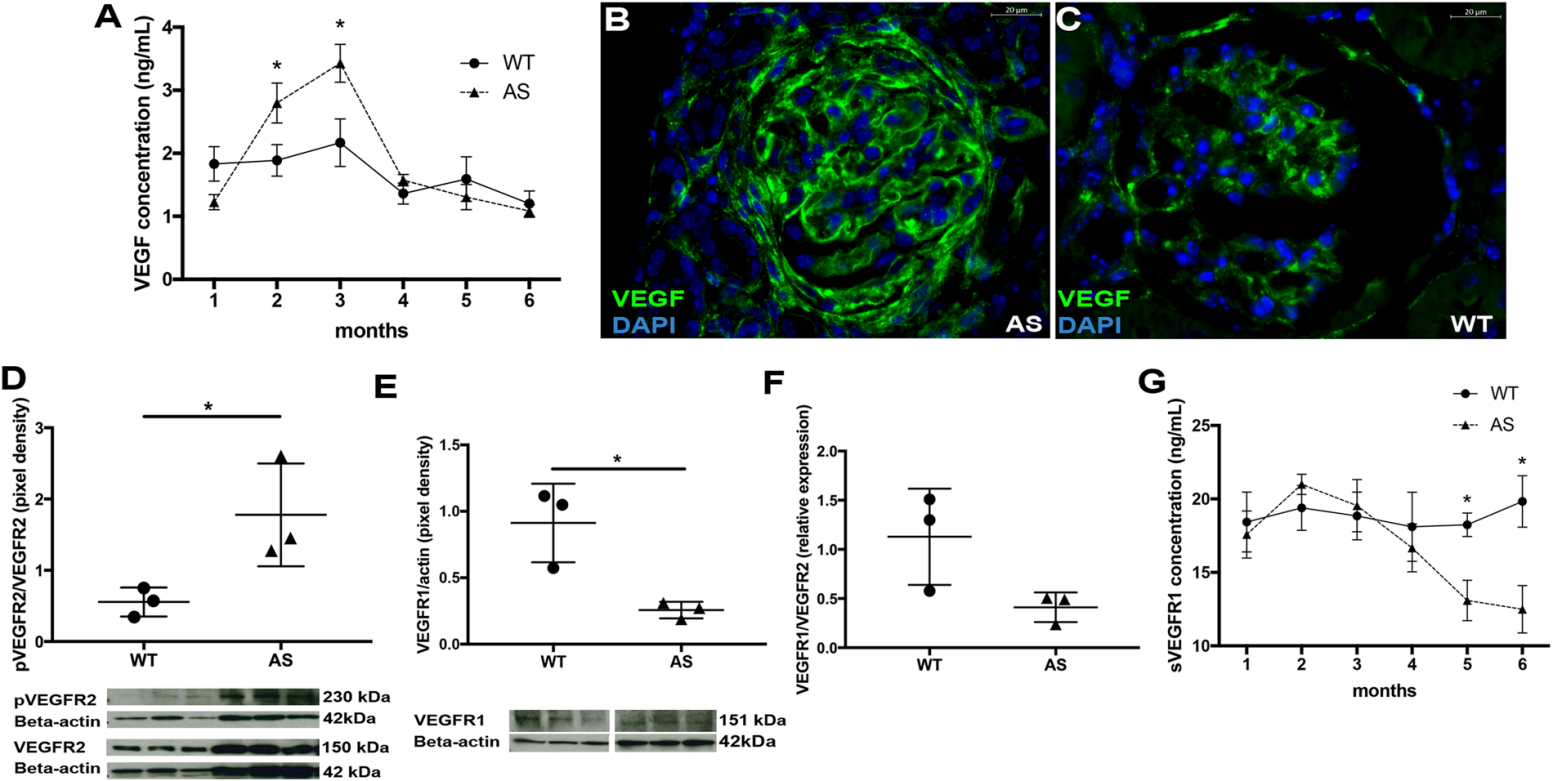
Evaluation of VEGF, pVEGFR2, VEGFR1 and sVEGFR1 expression within Alport glomeruli during disease progression. Changes in VEGF signaling activity were evaluated in glomeruli isolated from kidneys of WT and Alport mice over the course of disease progression from 1 to 6 months of age by ELISA, immunohistochemistry and Western Blot. As shown in the graph (**A**), glomerular VEGF, measured by ELISA, increased gradually during the early phase of disease peaking at 3 months as compared to time-matched siblings, and dropped thereafter (n = 4 WT mice/time point and n = 4 AS mice/time point). Representative VEGF staining at 3 months confirmed the presence of elevated VEGF expression (green) in Alport glomeruli (**B**, 40x) as compared against WT controls (**C**, 40x). Nuclear staining with DAPI (blue). Graphs representing immunoblot data of pVEGFR2/VEGFR2 ratio (**D**, 230 kDa/150 kDa), VEGFR1 expression (**E**, 151 kDa) and VEGFR1/VEFR2 ratio (**F**) in glomeruli of Alport mice (n = 3 AS mice) compared to their WT littermates (n = 3 WT mice) at 3 months of age. Imbalanced expression of VEGF signaling is confirmed by the increase of pVEGFR2/VEGFR2 ratio and decrease of VEGFR1 expression. Immunoblots were quantified by densitometry. All VEGFR1, VEGFR2 and pVEGFR2 measurements were normalized against corresponding housekeeping gene, β-actin, 42 kDa; pVEGFR2/VEGFR2 ratio was assessed after β-actin normalization. Glomerular sVEGFR1 expression measured by ELISA decreased in advanced Alport glomeruli (n = 4 AS mice/time point) as compared to time-matched siblings (n = 4 WT mice/time point) (**G**). Two-tail student *t*-test was used to determine differences between WT and AS mice. All values in (**A**) and (**G**) are presented as mean ± SEM. All scatter plot values are presented as mean ± SD; (*p < 0.05).

As shown in Fig. 1A–C, VEGF expression, mainly produced by podocytes [Suppl. Figure 1A–H], was markedly altered early on in disease and peaked at 3 months but returned to baseline level thereafter. VEGF over-activation was shown by the increased pVEGFR2/VEGFR2 ratio in AS glomeruli [Fig. 1D]. At 3 months of age VEGFR1 expression [Fig. 1E], and the VEGFR1/VEGFR2 ratio were significantly decreased in AS [Fig. 1F]. In addition, sVEGFR1 was significantly decreased in later stages (5–6 months of age) [Fig. 1G]. Of note, at 6 months of age, no major shifts in the VEGFR1 and VEGFR2 expression were detected between WT and AS glomeruli [Suppl. Figure 1I–K] likely indicating that glomerular cells might be counter-reacting to the VEGF signaling alteration at this advanced stage of AS by turning down the sensitivity to the signal.

### GEC characterization in Alport mice

**Figure 2:**
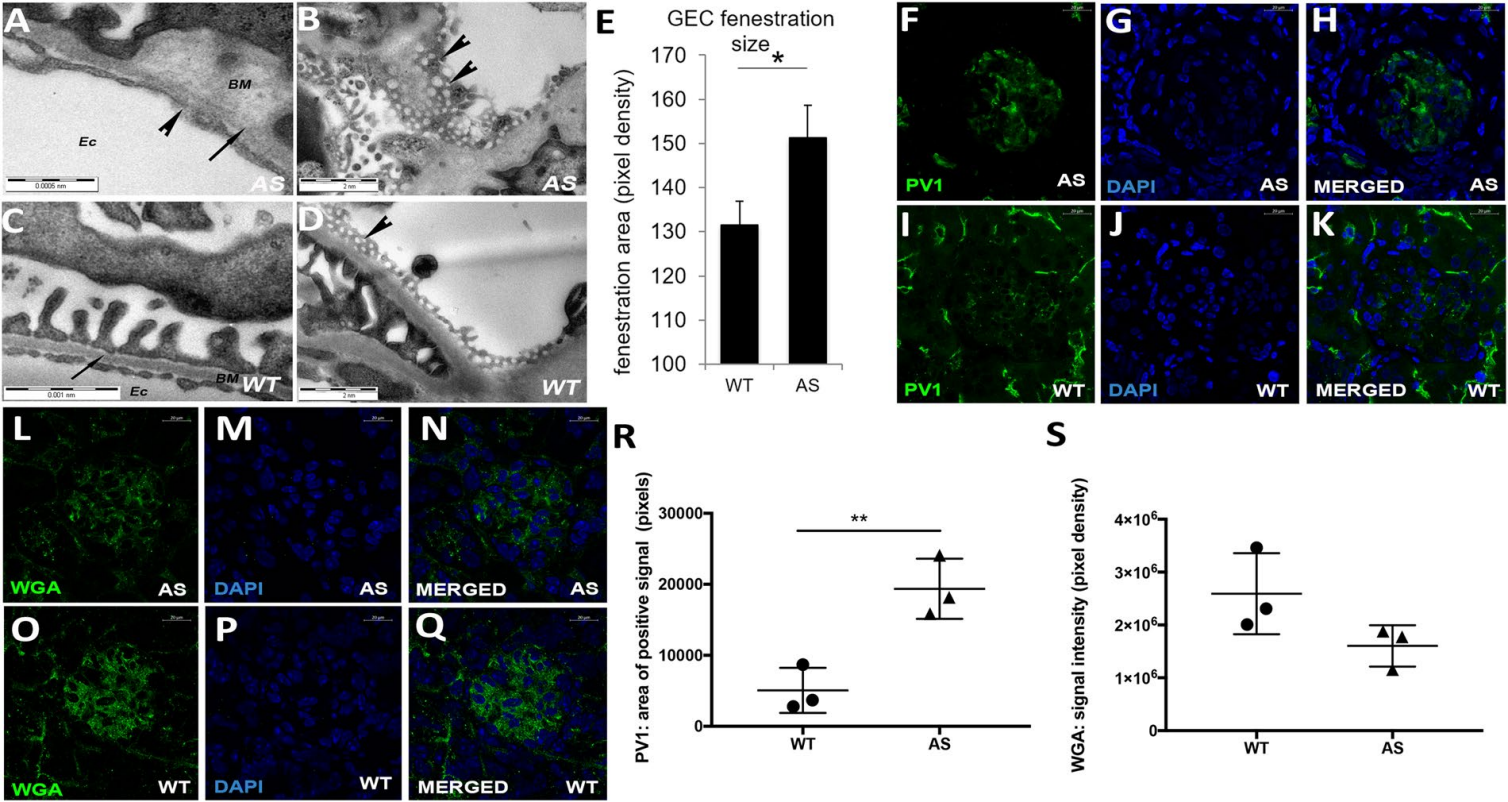
GEC damage in Alport mice. Alport mice showed GEC damage during early stage of disease (**A**–**S**). Representative transmission electron microscopy images showing characteristic thickening and splitting of the GBM in Alport mice (arrow, **A**, 28,000x), enlarged endothelial fenestrations and ruptured endothelial layer (arrowheads, **A–B**, 28,000x) when compared to their littermate WT mice (**C–D**, 28,000x). GEC fenestrations were enlarged in Alport mice (n = 3 AS mice) at 3 months of age when compared to the WT mice (n = 3 WT mice) as shown by the graph in **E**. Fenestration size was measured by ImageJ in 20 TEM images per sample. Values are presented as mean ± SEM. Representative confocal immunofluorescence images of AS glomeruli showing altered PV1 (green, **F–H**, 63X) and WGA (red, **L–N**, 63X) expression compared to WT glomeruli (**I**–**K**, **O**–**Q**, 63X) at 3 months of age (n = 3 AS mice and n = 3 WT mice). Of note, PV1 staining is present in peritubular capillaries that unlike GEC express normal level of PV1 under physiological conditions. Nuclear staining with DAPI (blue). Graphs representing morphometric quantification of the PV1 (**R**) and WGA staining (**S**) in AS and WT glomeruli. Measurements were performed by ImageJ software (NIH) and each dot represents the median value of 20 images per mouse. Two-tail student *t*-test was used to determine differences between WT and AS mice, (*p < 0.05, **p < 0.01). All scatter plot values are presented as mean ± SD; (*p < 0.05).

We next examined the impact of VEGF modulation on GEC. TEM scans showed characteristic splitting of the GBM consistent with AS pathology, whereas WT mice had normal basement membrane thickness [Fig. 2A,C, arrows]. In addition, GEC presented an altered morphology characterized by disruption of fenestrations when compared to WT [Fig. 2A–D, arrowheads] at 3 months of age. Fenestration size was significantly increased in Alport mice further confirming changes in GEC morphology [Fig. 2B,D,E] during VEGF peak expression. In addition, AS glomeruli showed *de novo* expression of plasmalemma vesicle associated protein-1 (PV1, which plays a key role in diaphragm formation in fenestrae and maintenance of endothelial integrity^16,17^, [Fig. 2F–K,R]) and alteration of the glycocalyx as shown by downregulation of the wheat germ agglutinin (WGA) expression [Fig. 2L–Q,S] during the VEGF peak^18,19^.

**Figure 3:**
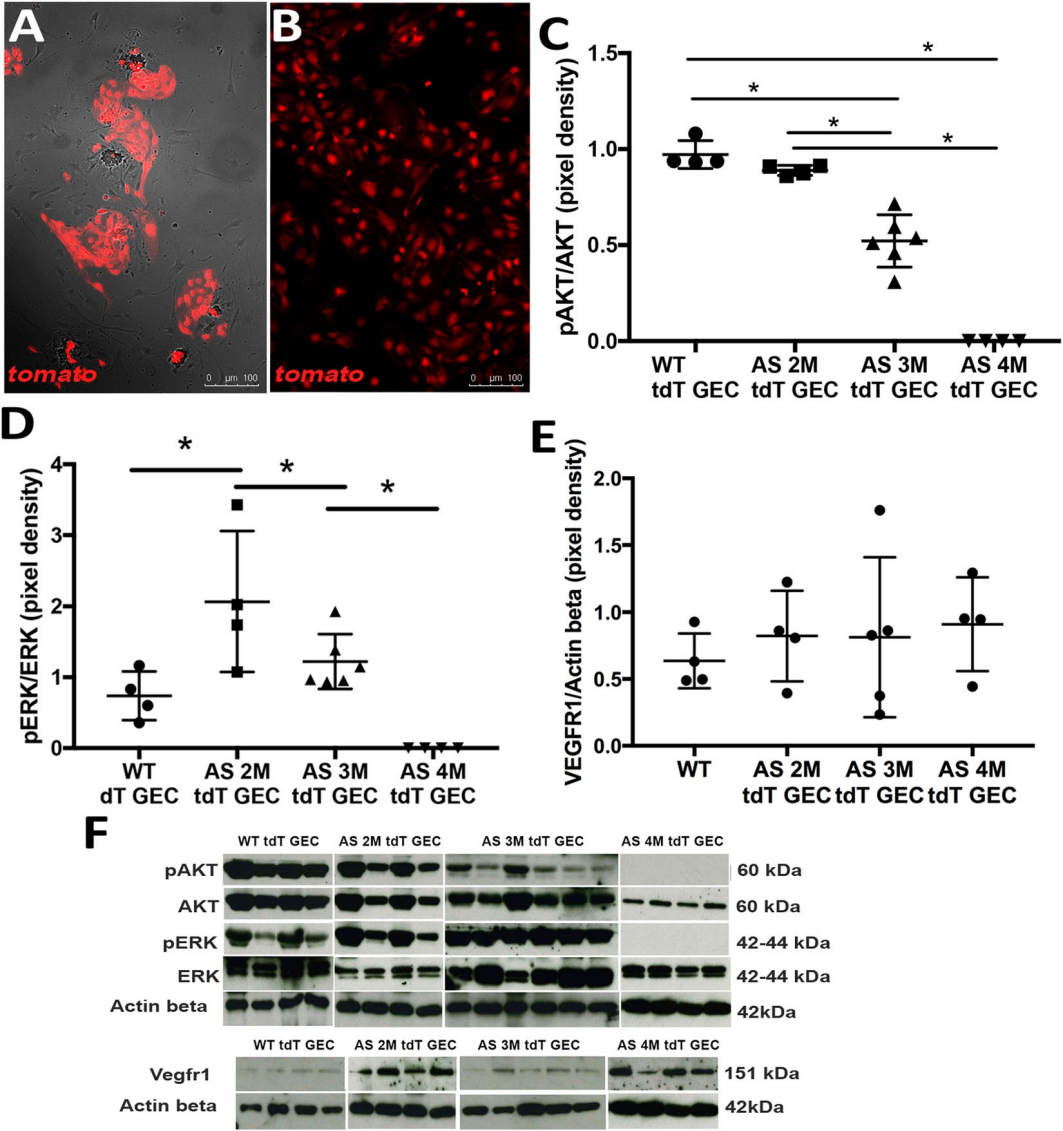
VEGF signaling is altered in GEC isolated from Alport mice. VEGF signaling changes in GEC were studied using tdTomato-labeled GEC (*tdT* GEC) isolated by FACS from Alport-Tek^*tdT*^ glomeruli (**A**, 10x). *tdT* signal (red) is strongly present in all cells as shown in (**B**) (10x, two passages in culture) (n = 3). Graphs representing immunoblot data of pAKT/AKT (**C**, 60 kDa/60 kDa), pERK/ERK (**D**, 42 kDa/42 kDa), VEGFR1 expression (**E**, 151 kDa) in glomeruli of *tdT* GEC derived from WT mice and Alport-Tek^*tdT*^ mice at different ages. Imbalanced VEGF signaling is evident during disease progression in GEC, identified by a strong alteration in pAKT/AKT and pERK/ERK signaling downstream of VEGFR2. (**F**) Immunoblots of all the experimental groups used to generate the graphs presented in Figures **C**–**E**. Immunoblots were quantified by densitometry (VEGFR1 measurements were normalized against corresponding housekeeping gene, β-actin, 42 kDa). These data were obtained using GEC derived from n = 4 WT at 5 months of age, n = 4 AS mice at 2 months of age, n = 6 AS mice at 3 month of age, n = 4 AS mice at 6 months of age. One-way ANOVA with Tukey’s *post hoc* test was used to analyze the data and scatter plot values are presented as mean ± SD (*p < 0.05).

To specifically study the correlation between VEGF modulation and GEC damage, we generated an Alport mouse with fluorescently labeled GEC (Alport-Tek^*tdT*^), among other endothelial cells in other organs. We confirmed that glomeruli from WT-Tek^*tdT*^ mice present with a strong tdTomato (tdT) signal (in red) in GEC [Suppl. Figure 2A–E, Fig. 3A,B]. Of note, we identified two distinct populations of tdT positive cells within the glomerulus, represented with bright and dim tdT expression [Suppl. Figure 2E,J,K]. The FACS sorted bright tdT-GEC cells were further confirmed for their endothelial phenotype for expression of CD31 by RT-PCR [Suppl. Figure 2F] for VE-Cadherin by flow cytometry [Suppl. Figure 3B] and for CD31 and Tie-2 by immunostaining [Suppl. Figure 3C]. The tdT-GEC were negative for markers like PDGFRβ, WT1 and Nephrin [Suppl. Figures 2G,H and 3A,D,E]; thus indicating that within the glomerulus, the tdT is expressed exclusively in GEC, allowing us to isolate GEC free from contamination of other glomerular cells. The dim population lacked strong expression of endothelial markers [Suppl. Figure 2I]. Therefore for the purpose of this study, we used the former in all the experiments described.

**Figure 4:**
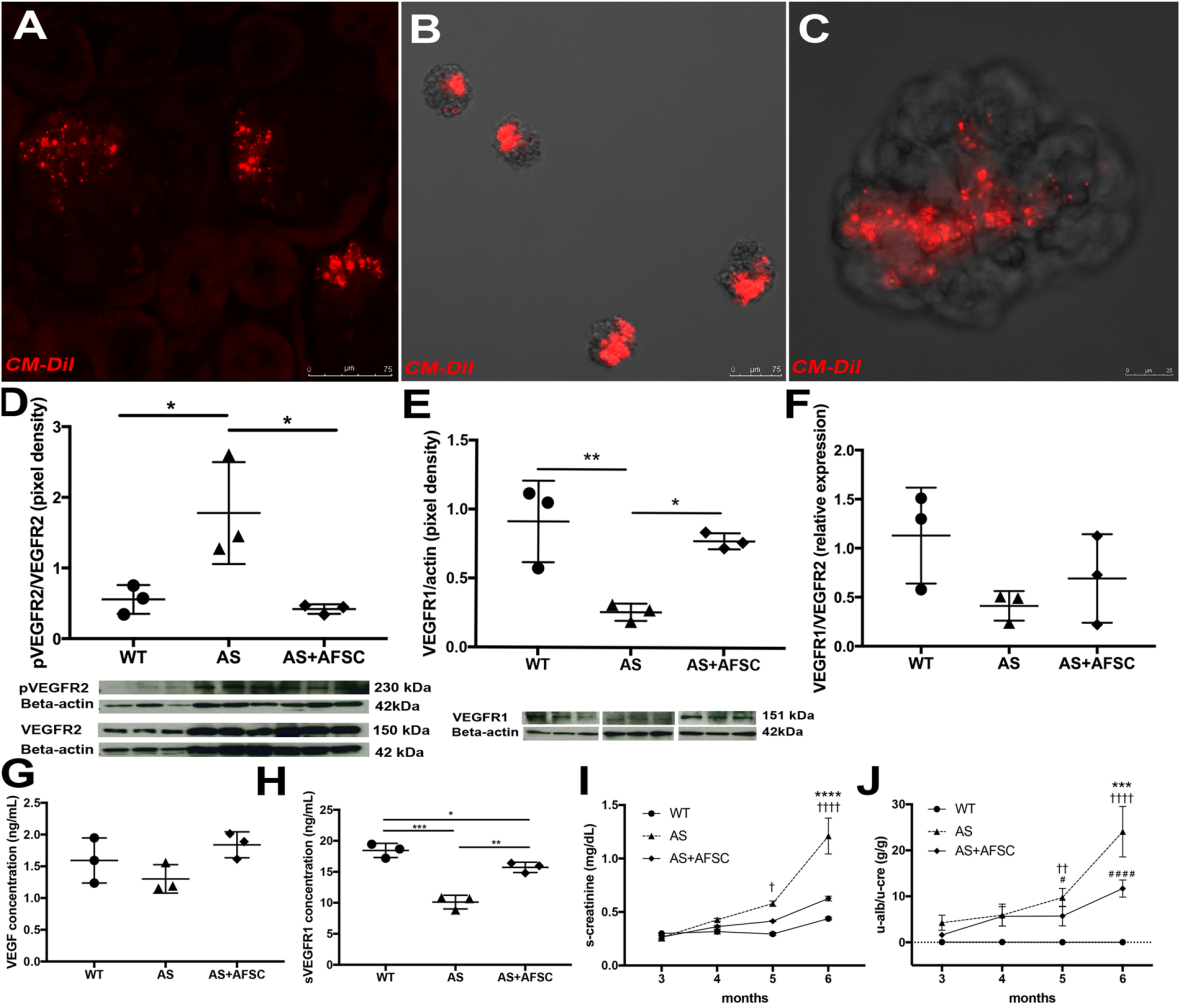
AFSC homing to the kidney and modulation of VEGF, pVEGFR2, VEGFR1 and sVEGFR1. Alport mice were injected at 3 months of age to study homing of AFSC and modulation of VEGF signaling. Injected labeled-AFSC (CM-DiI, red) were traceable after 5 days and could be detected by fluorescence within Alport glomeruli (**A**, 20x). Representative image of isolated glomeruli from Alport mice after 5 days of injection showing CM-DiI labeled AFSC in red (**B**, 10x; **C**, higher magnification 40x). Injection of AFSC modulated expression of pVEGFR2/VEGFR2 ratio (**D**, 230 KDa/150 kDa), VEGFR1 (**E**, **1**51 KDa) and VEGFR1/VEGFR2 ratio (**F**) within the glomeruli of Alport mice (n = 3 AS mice) compared to non-injected siblings (n = 3 AS mice) after 2 weeks of injection, restoring the activity of these VEGFRs almost at normal levels. Immunoblots were quantified by densitometry (VEGFR1, VEGFR2 and pVEGFR2 measurements were normalized against corresponding housekeeping gene, β-actin, 42 KDa; pVEGFR2/VEGFR2 ratio was assessed after β-actin normalization). As shown in graph **G–H**, injection of AFSC modulated levels of VEGF and sVEGFR1 as measured by ELISA in Alport (n = 3 AS mice) versus non-treated mice (n = 3 AS mice) at 7 weeks after injection. Injection of AFSC also ameliorated serum creatinine (**I**) and proteinuria (**J**) in Alport mice (n = 6) compared to non-treated Alport mice (n = 6) and WT (n = 5) measured over 12 weeks period after AFSC injection. ^#^Significant difference between WT and AS+AFSC; ^†^Significant difference between WT and AS. *Significant difference between AS and AS+AFSC. One-way ANOVA with Tukey’s *post hoc* test was used to analyze the data in Fig. (**D**–**H**); Two-way ANOVA with Tukey’s *post hoc* test was used to analyze the data in Fig. (**I,J**); values in (**I**) and (**J**) are presented as mean ± SEM. All scatter plot values are presented as mean ± SD; (*p < 0.05, **p < 0.01, ***p < 0.001).

Isolated GEC from Alport-Tek^*tdT*^ presented altered expression of VEGF signaling, including pAKT and pERK (downstream of VEGFR2). The levels of these downstream markers varied largely along disease progression with values close to normal at 2 months of age and almost absent at 4 months of age [Fig. 3C,D]; no significant variation in VEGFR1 expression was detected [Fig. 3E,F]. These variations of the VEGF signaling in GEC present a trend similar to that observed within the glomeruli specifically in relation to the turning down of the signaling after the VEGF peak. Functionally, these variations correspond to the onset of serum creatinine and proteinuria in AS mice as shown in Fig. 4I,J.

### AFSC effects on intraglomerular regulation of VEGF signaling and on GEC biology

To study the modulation of VEGF signaling by AFSC, mice were treated with a single injection of CM-DiI or Q-Dot tagged AFSC (the same cell line as published^12^) prior to the onset of a high level of proteinuria [Suppl. Figure 4A,B]. AFSC localized predominantly within the kidney, specifically in glomeruli [Fig. 4A–C and Suppl. Figure 4C,D] in close association with GEC as shown by a co-staining with VE-cadherin (endothelial cells) and CD2AP (podocytes) [Suppl. Figure 4D] after 5 days of delivery. Cells were not found in other organs in any significant number [Suppl. Figure 4E,F], as previously reported^12^. After 2 weeks of AFSC injection, the pVEGFR2/VEGFR2 ratio and VEGFR1 expression within the isolated glomeruli was comparable with that of the WT [Fig. 4D,E]. Although no significant changes in the VEGFR1/VEGFR2 ratio was observed between AFSC treated and non-treated animals, AFSC were able to significantly reduce VEGFR2 phosphorylation when total VEGFR2 expression remained unchanged [Fig. 4D]. After 7 weeks of injection, VEGF level was unchanged and sVEGFR1 expression was elevated and was comparable with that of the WT [Fig. 4G,H], followed by mitigation of serum creatinine and proteinuria in treated mice [Fig. 4I,J]. To verify that AFSC can mitigate GEC damage, we performed co-culture assays between AFSC and GEC (with no cell-cell contact) overexposed to VEGF for 24 h. Exposure to VEGF at 100ng/ml caused increased expression of VEGF, pVEGFR2 in GEC [Suppl. Figure 5A,B]. After 24 hr of VEGF exposure, GEC demonstrated changes in expression pattern of VE-cadherin and CD31 [Suppl. Figure 5C–F] and decreased cellular proliferation that was rescued by AFSC [Suppl. Figure 5G–J]. AFSC co-culture normalized increased expression of genes like VEGF, fibronectin, and Ccl5 [Suppl. Figure 5K], thus protecting GEC from VEGF-induced damage.

### Characterization of AFSC-derived EVs

**Figure 5:**
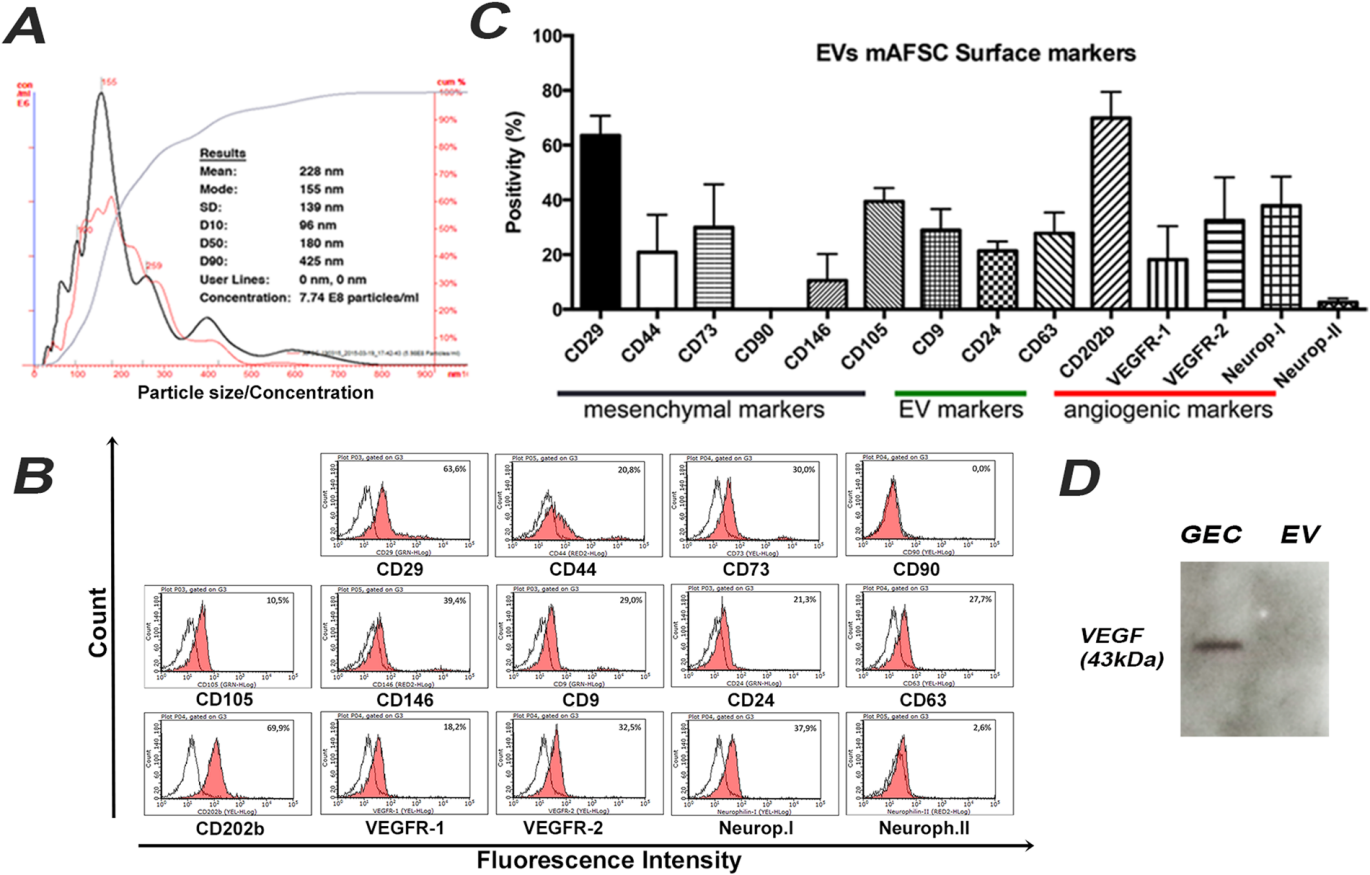
AFSC-EV characterization. (**A**) Graph representing Nanosight analysis of AFSC-derived EVs. The mean size and particle concentration are calculated by the Nanoparticle Tracking Analysis software. (**B**) FACS analyses of AFSC-derived EVs showing the expression of mesenchymal (CD29, CD44, CD73, CD90, CD105 and CD146), exosomal (CD9, CD24 and CD63) and angiogenic related (CD202b, VEGFR1, VEGFR2, Neuropilin-I and Neuropilin-II) surface markers. EVs were derived from the same cell line and the FACS analysis was repeated in quadruplicate. Dot lines indicate the isotopic controls, filled area the specific antibodies. (**C**) Representative FACS graphs related to figure (**B**). (**D**) Representative Western Blotting showing absence of VEGF (43 KDa, dimer) within AFSC-derived EVs compared to GEC used as control. Values are presented as mean ± SEM.

Since EVs have been established as an important mechanism of cell-to-cell communication^13–15^ we hypothesized that secretion of EVs from AFSC could be one of their mechanisms of action. In order to test our hypothesis, EVs from culture supernatants of AFSC were isolated by sequential ultracentrifugation. By Nanosight analysis, EVs were characterized as a heterogeneous population ranging from 100 nm to 400 nm in size, and their production in basal culture conditions appeared as roughly 2 × 10^10^ EVs released by million cells in 24 hours [Fig. 5A]. AFSC-derived EVs expressed surface markers typical of the cell of origin (CD73 and CD29) and CD24, a marker of amniotic fluid-derived exosomes^20^ [Fig. 5B,C]. EVs also expressed VEGFR1 and VEGFR2 [Fig. 5B,C] but did not contain VEGF [Fig. 5D]. EVs contained miRNAs that specifically modulate VEGF/VEGFRs signaling^21^ including miR-16.1, miR-23a, miR-27a, miR-93, miR-221, miR-145 and miR-322 [Suppl. Figure 6A].

To demonstrate that AFSC-derived EVs can be transferred to GEC, AFSC were transduced with LentiBrite GFP packaged lentiviral particles to constitutively express GFP and additionally tagged with CM-DiI. After overnight incubation with GECs, red/green EVs were found within the cytoplasm of GEC in proximity to the perinuclear zone [Suppl. Figure 6B–I].

### AFSC-derived EVs contribute to VEGF signaling modulation in GEC

**Figure 6:**
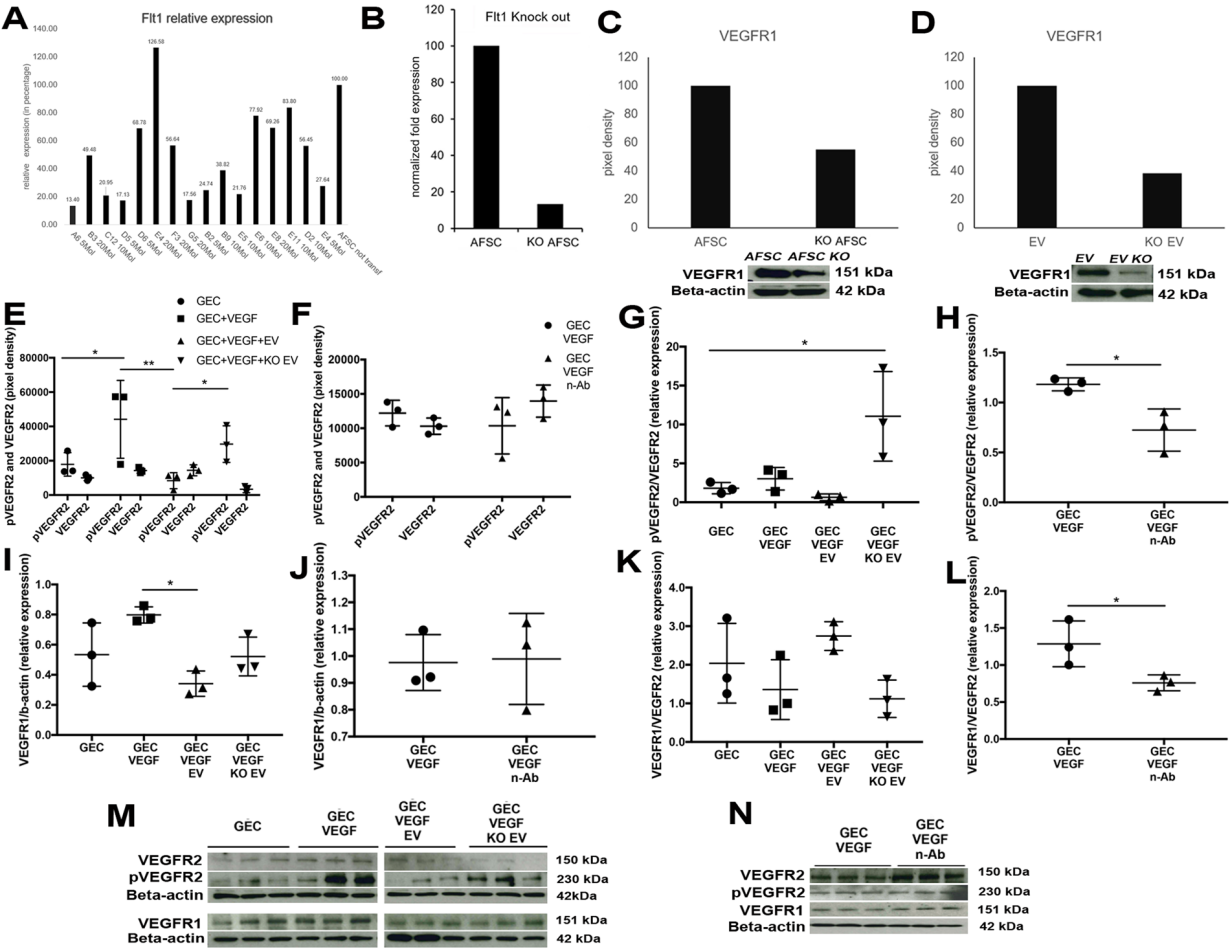
Generation of AFSC/EV^*Flt1*−/−^ and VEGFR2 and VEGFR1 modulation by AFSC-EVs in GEC. (**A**) Different viable clones obtained from AFSC transduced with Flt1 shRNA lentiviral particles where analyzed by qPCR for Flt1 expression after puromycin selection. AFSC were transduced with different titrations of viral particles (5, 10, 20 MOI) to obtain the best transduction efficiency. As shown, resulting puromycin-resistant clones have varying levels of Flt1 expression due to the random integration of the lentiviral construct into the genome of the cells. Clone A6 (first column) was selected as the most efficient. (**B**) Relative gene expression of Flt1 in non transduced AFSC compared to Flt1 KO AFSC (clone A6) showing 90% reduction. VEGFR1 protein (151 KDa) expression was quantified by Western Blot analysis in cell lysates of AFSC and KO AFSC (clone A6) (**C**) and EVs isolated from both cell populations (**D**) showing respectively 50% and 60% reduction. All measurements were normalized against their corresponding housekeeping gene, β-actin, 42 kDa. (**E–L**) Graphs showing differential expression of pVEGFR2 and VEGFR2 (**E**, 230 KDa and 150 KDa), pVEGFR2/VEGFR2 ratio (**G**), VEGFR1 expression (**I**, 15 KDa) and VEGFR1/VEGFR2 ratio (**K**) in GEC (basal condition, control), GEC stimulated with VEGF (100 ng/ml) and treated with EVs and KO EVs for 24 hr. Expression of pVEGFR2 and VEGFR2 (**F**), pVEGFR2/VEGFR2 ratio (**H**), VEGFR1 expression (**J**) and VEGFR1/VEGFR2 ratio (**L**) were also evaluated in GEC treated with neutralizing VEGF antibody (used as control). Both pVEGFR2 and VEGFR1 were restored to basal level in the presence of normal EVs and of the neutralizing VEGF antibody, but not in the presence of KO EV. Experiments were repeated in triplicate and Western Blot bands are presented in (**M)** and (**N)**. Immunoblots were quantified by densitometry (VEGFR1, measurements were normalized against their corresponding housekeeping gene, β-actin, 42 KDa). One-way ANOVA with Tukey’s *post hoc* test was used to analyze the data between 3 or more groups. All scatter plot values are presented as mean ± SD, (*p < 0.05).

To demonstrate EV ability to modulate VEGF activity and confirm the potential role of VEGFR1 and sVEGFR1 in GEC damage/repair processes we generated knockout (KO) EVs for Flt1, EV^*Flt1*−/−^, (Flt1 gene codifies for both the VEGFR1 and sVEGFR1) by transfecting AFSC using a shRNA Lentiviral Particles Transduction system. The clone with the highest efficiency of transduction was used to derive and collect EV^*Flt1*−/−^. Both, KO AFSC and derived EVs showed reduced VEGFR1 expression [Fig. 6A–D]. After generation of the EV^*Flt1*−/−^ we exposed GEC to VEGF and treated them with normal EVs, KO EVs and neutralizing VEGF antibody (used as a positive control). pVEGFR2/VEGFR2 ratio and VEGFR1 expression were restored to baseline in the presence of EVs [Fig. 6E,G,I,K] but not with KO EVs, thus confirming that sVEGFR1 plays a role in regulating VEGFR1 expression and VEGFR2 activity. Importantly, EVs seem to have more efficiently downregulated VEGFR2 phosphorylation when compared with neutralizing antibody [Fig. 6E,F], and they both showed a similar effect when assessed against total VEGFR2 [Fig. 6G,H]. Interestingly, EVs but not anti-VEGF neutralizing antibody downregulated VEGFR1 expression in GEC [Fig. 6I,J]. We speculate that EVs might present multiple mechanisms of action (due to their cargo) that are lacking in the control antibody experiment. Of note, slight differences in VEGFR1 level between *in vitro* and *in vivo* data can be attributed to the fact that *in vivo* data shows expression of all the glomerular cell types, including GEC, whereas *in vitro* data only reflects GEC response.

**Figure 7:**
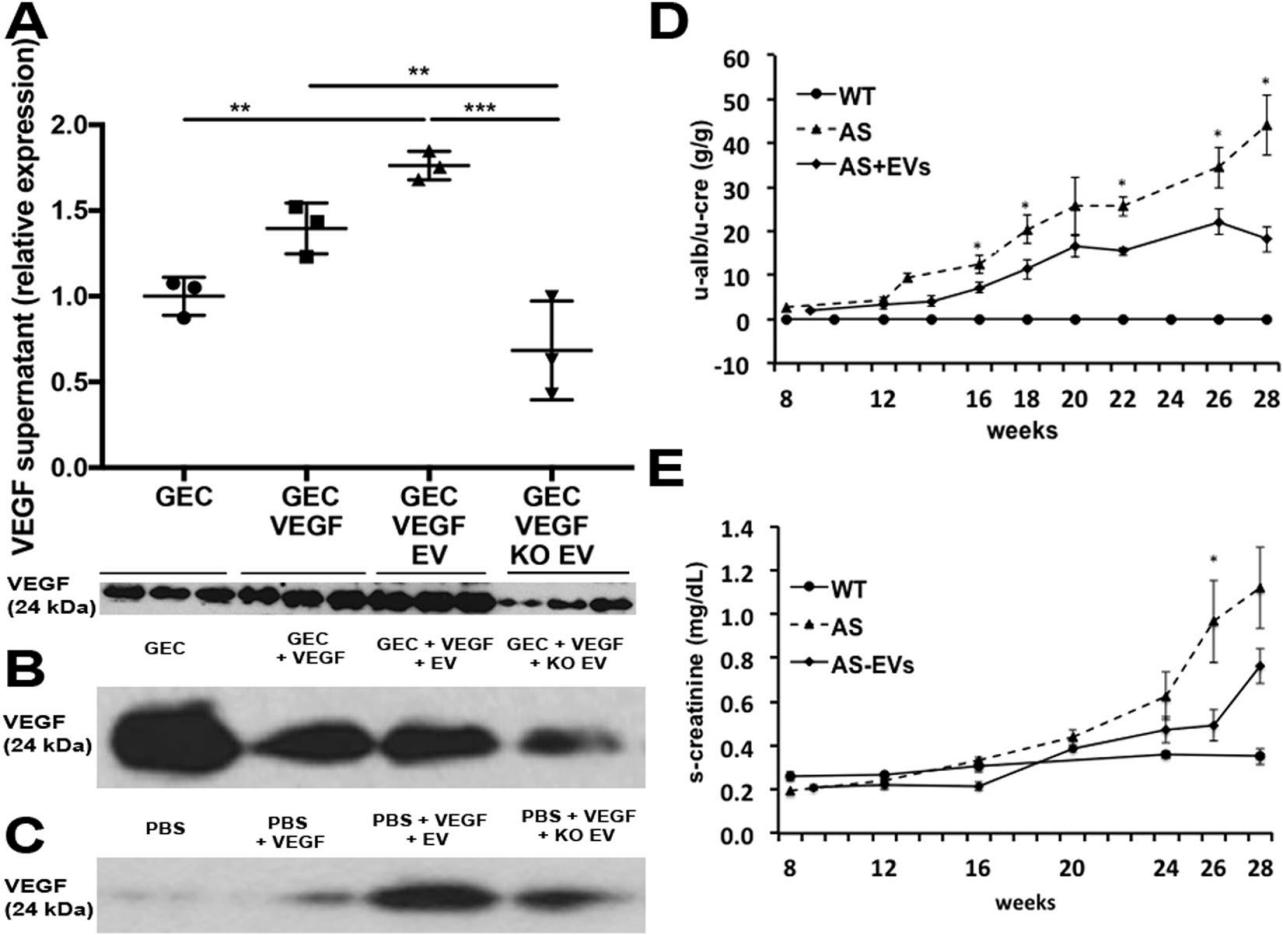
VEGF modulation by AFSC-EVs in GEC and AFSC-EV effect on renal function. (**A**) Graph showing level of VEGF (24 KDa, monomer) within the media collected from GEC (basal condition, control), GEC stimulated with VEGF (100 ng/ml) and treated with EVs and KO EVs for 24 hr. VEGF level was higher in GEC stimulated with normal EVs compared to GEC stimulated with KO EV. Experiments were repeated in triplicate and data were quantified (measurements were normalized against VEGF basal level in GEC only group since Western Blot was performed in collected media; Western Blot bands are presented below the graph). (**B**) Representative immunoblot of VEGF after co-immunoprecipitation with VEGFR1 on the supernatant collected from experimental groups described in (**A**), showing the inefficiency of the KO EV to trap VEGF when compared to that of normal EVs. (**C**) Representative immunoblot of VEGF, after co-immunoprecipitation with VEGFR1 on samples of PBS solution containing high VEGF dose (100ng/ml) and treated with EVs and KO EVs for 24 hr further confirming the inability of the KO EV to effectively trap VEGF compared to normal EVs. The weak band detected in the PBS/VEGF only group represent a VEGF carryover due to incomplete removal of VEGF during the washing steps. Immunoblots were quantified by densitometry. (**D**) Injection of EVs ameliorated proteinuria in Alport mice (n = 8) compared to non-treated Alport mice (n = 8) and WT (n = 5) measured over 28 weeks period after EVs injection. (**E**) EV treatment also improved serum creatinine level within the same treated animals. Note: toward the end of the study at 28 weeks few mice were lost due to advanced disease. *Significant difference between AS and AS + AFSC. One-way ANOVA with Tukey’s *post hoc* test was used to analyze the data. All scatter plot values are presented as mean ± SD, (*p < 0.05, **p < 0.01, ***p < 0.001).

Interestingly, GEC exposed to EVs showed a greater retention of VEGF within the media [Fig. 7A] compared to GEC exposed to KO EV. These data suggest that EVs (that do not contain VEGF as reported above) could trap excess VEGF contained in the media, thus preventing its binding to VEGFRs on GEC and internalization subsequently regulating VEGFR2 and VEGFR1 activity. To prove our hypothesis we performed a co-immunoprecipitation assay, using anti-VEGFR1 antibody to pull down VEGFR1 on the same culture media and probed for VEGF. Indeed, Fig. 7B shows a lower level of VEGF in the experimental group with KO EVs compared to normal EVs. This data was confirmed independently using a different assay [Fig. 7C], where EVs and KO EVs were exposed to VEGF without the presence of GEC. Both these experiments confirmed that EVs can trap VEGF through VEGFR1, thus balancing VEGF signaling.

Direct administration of EVs into AS mice at 8 weeks of age and before the onset of a high level of proteinuria improved renal physiological parameters, including proteinuria and serum creatinine [Fig. 7D,E]. In particular, proteinuria level was significantly ameliorated in treated animals over many weeks as compared to non-treated mice [Fig. 7D].

## Discussion

Various molecular signaling pathways contribute to the cell-cell communication between podocytes and GEC^1,22^ but VEGF expression plays a key role in the maintenance of the structure and function of glomerular capillaries including permeability, while its alteration plays a major role in CKD^5^. In normal animals, the blockade of VEGF correlates with GEC damage and an increase of proteinuria^23^. On the contrary, in diabetic mice, VEGF is upregulated, and its blockade ameliorates diabetic albuminuria^24^. Therefore, both deficiency and excess of VEGF appear to be detrimental to the physiological integrity of glomerular capillaries. An imbalance between VEGF and sVEGFR1 has been reported in many diseases, including the kidney^6–9^, as inducing widespread endothelial dysfunction, proteinuria, and hypertension.

Importantly, accumulation of VEGF in human glomeruli was reported in various kidney diseases^25,26^, including AS^27^, thus supporting its importance in kidney disease. However, the biological role of VEGFR1/sVEGFR1 in the context of glomerular endothelial damage and its correlation with increased proteinuria is still unknown. Because GEC express an abundance of both VEGFR2 and VEGFR1, it is plausible that VEGFR1 and sVEGFR1 might have key importance in the regulation of the VEGF signaling activity in these cells.

Indeed, in our CKD animal model, we found that VEGF is elevated particularly early in the disease, reaching the maximum expression at 3 months of age, and then decreasing in more advanced stages. Interestingly, the level of sVEGFR1 within AS glomeruli also decreased after the peak of VEGF expression, thus possibly indicating that sVEGFR1 fails to effectively counter-balance the VEGF increase gradually causing glomerular damage. In an elegant study, Quaggin’s group^28^ identified that podocytes produce sVEGFR1 and that its elimination in podocytes causes profound cellular damage and increased proteinuria. Since podocytes are the major producers of VEGF^2,3^ within the glomerulus and since sVEGFR1 has autocrine activity on podocytes, a possible explanation for decreased VEGF and sVEGFR1 levels with disease progression might be the loss of podocytes during AS progression. Indeed, we showed^12^ that AS glomeruli at 4 months of age have a significantly reduced number of podocytes compared to WT at the same age.

Significant changes in VEGFR2 activation and VEGFR1 expression were evident during the initial phase of AS progression when podocyte loss is not predominant and proteinuria is still low. These observations strongly suggest that in addition to the podocyte, which has been considered as the culprit of CKD progression including AS^29^, the endothelium could also contribute to the initiation and progression of glomerular damage in AS as well as potentially in other forms of CKD.

Indeed, GEC damage represented by disruption of fenestration size and glycocalyx structure is very evident in 3 months old kidneys. We also confirmed that GEC at this stage present with abnormal activation of AKT and ERK, especially during and after the VEGF peak. Therefore, we speculate that in early stages of AS, the elevated VEGF activity might be fundamental in promoting GEC damage, thus, in turn, causing podocyte injury and proteinuria. Interventions aimed at preventing early endothelial damage, possibly by regulating VEGF expression might, therefore, present an alternative pathway to delaying renal disease progression.

We have previously shown that AFSC, when injected before the onset of proteinuria, are able to slow down disease progression in AS mice^12^. Here we demonstrate that AFSC home predominantly within the glomerulus of Alport mice and localize adjacent to the GEC, thus potentially triggering a regulatory response in GEC. Thus, AFSC decreased pVEGFR2 and increased VEGFR1 within the glomeruli, confirming that AFSC can modulate VEGF/VEGFRs activity. We also previously reported that AFSC injection preserves podocyte number^12^, which might account for the normalization of VEGF and sVEGFR1 levels observed in injected mice, as presented herein. Preservation of podocyte number and regulation of VEGF and sVEGFR1 expression were also correlated with improvement of kidney function.

Due to their close proximity to GEC, we hypothesized that one of the possible mechanisms of renoprotection by AFSC could be via EV release. Recent studies have demonstrated that EVs released from cells are an integral component of the cell-to-cell communication network involved in tissue regeneration^13–15^ and therefore may contribute to the paracrine activation of stem cells in renal regeneration, by directly activating target cells to secrete functionally active agents^30^.

Several groups, including us, have already demonstrated the *in vivo* ability of EVs derived from bone marrow mesenchymal stem cells to provide protection from acute kidney injury^31–34^. Here, we demonstrate that indeed AFSC do secrete EVs, which express surface mesenchymal/exosomal markers as well as a number of angiogenic receptors, including VEGFRs. Importantly, we demonstrated for the first time that administration of AFSC derived EVs can provide a functional benefit, similar to that of injection of AFSC. This effect could be due to EVs functioning as a “trap” for excess VEGF by binding to VEGFR1 presented on the EVs surface. We, therefore, speculate that modulation of VEGF within the glomeruli involves trapping of VEGF by VEGFR1 on the surface of AFSC-derived EVs. In this context, the EVs presenting VEGFR1 on their surface might counterbalance the decrease in sVEGFR1 observed during AS progression. It is also known that VEGF signaling disruption is a major occurrence in diabetic nephropathy, especially during the early phase of the disease^5^. Therefore, we speculate that EVs might also regulate VEGF expression in diabetic nephropathy as well as in other forms of CKD with altered glomerular VEGF signaling. Importantly, despite the higher level of VEGFR2 over VEGFR1 on EVs, the affinity of the VEGF-VEGFR1 interaction is ten times stronger to that between VEGF-VEGFR2^35^. This mechanism of action could potentially be expanded to other molecules (such as TGFβ or ang II), since EVs present many different receptors. Downregulation of these molecules through “trapping” could thus be beneficial to resolving glomerular damage.

Recently, it was shown that EVs derived from AFSC contain specific miRNAs as cargo^36,37^. Indeed, we also found that EVs contain miRs known to act in the modulation of VEGF levels (miR-16.1, -93), VEGF receptors (miR-16.1), as well as both positive and negative regulators of the VEGF signal transduction cascade (miR-23a, -27a, -221, -322 and -145)^21^. This angiomodulatory cargo could involve potential new mechanisms of VEGF regulation, by triggering mechanisms of repair/regeneration at the transcriptional level. We focused on the VEGF signaling because of its importance in maintenance of the glomerular capillary network. Moreover, the presence of a wide variety of surface markers as well as regulatory miRNAs within the EV, likely indicates potential of the AFSC-derived EVs to be able to regulate other important mechanisms essential to the glomerular homeostasis, and especially to the endothelium (such as the oxidative stress pathway, iNOS, TGF-β, and Ang II). However this tantalizing finding requires further investigation and is beyond the scope of the current study.

Although in depth analyses would be required to determine if EVs account for all of the reno-protective effects of AFSC, we clearly demonstrated that EVs are capable of changing the course of disease progression in Alport mice (as evidenced by the regulation of proteinuria and serum creatinine), similar to that injected with AFSC. In conclusion, we demonstrated that 1. VEGF signaling plays an important role in the disruption of glomerular homeostasis and that injection of AFSC can restore the activity of this signaling pathway, possibly preventing further loss of kidney function. 2. Alterations in VEGF signaling affect the glomerular endothelium during early AS and before massive loss of podocytes and elevated proteinuria. 3. AFSC home within glomeruli in proximity to GEC and release “angiomodulatory” EVs. 4. AFSC-derived EVs can modulate VEGF signaling by podocytes and other glomerular cells (such as mesangial cells that can contribute to the variation of VEGF during disease progression) by trapping excess VEGF thorough VEGFR1-binding.

Since AFSC localize within the glomeruli in our animal model, we speculate that the delivery of EVs by AFSC can specifically target VEGF signaling in this key renal compartment, thus favoring a correctly dosed, local therapeutic action of VEGF exactly where it is needed. Potential strategies to modulate glomerular cell crosstalk, specifically for preventing initiation and progression of GEC damage will strengthen the rationale for novel therapeutic approaches directed towards homeostatic regulation of glomerular function.

## Methods

### AFSC culture, GFP infection and Flt1 knockout experiments

Clonal lines of AFSC were derived and cultured as previously described^12^. For GFP transfection, AFSC were seeded at a density of 2.5 × 10^4^/cm^2^ and transduced with LentiBrite GFP packaged lentiviral particles (Millipore, #17-10387) at 35MOI. Transduction efficiency was assessed by inverted fluorescence microscope (Leica DMI6000 B). AFSC^*GFP*^ were selected by FACS (BD, FACSAria III) and used for EV isolation.

AFSC^*Flt1*−/−^ were generated by using a shRNA Lentiviral Particles Transduction system (Santa Cruz Biotechnology, #sc-35395-V) following manufacturers’ instructions. Seeded AFSC (10,000/cm^2^) were transduced at 5MOI, and stable clones were selected via puromycin selection (10 ug/ml) for about 2 weeks. The clone with the highest KO efficiency was used for collecting EVs.

### Animal models (WT mice, Alport mice, WT- Alport-Tek^tdT^, Alport-Tek^tdT^), AFSC and EV injections and AFSC *in vivo* tracking, serum creatinine, and proteinuria measurements

All animals were purchased from the Jackson Laboratory^12^. The Alport-Tek^*tdT*^ mice were generated by breeding Alport mice (B6.Cg-Col4α5tm1Yseg/J) with an endothelial specific Cre-driver mouse (B6.Cg-Tg(Tek-cre)1Ywa/J and a tdT-reporter mouse (B6.Cg-Gt(ROSA)26Sor^tm14(Cag-td-Tomato)Hze^/J); these mice express tdTomato (*tdT*) in all endothelial cells including GEC. To study disease progression n = 24 WT and n = 24 Alport mice were sacrificed at 1, 2, 3, 4, 5 and 6 months of age (n = 4/time point) to isolate glomeruli. For TEM analysis n = 3 of WT and n = 3 of Alport mice were used. For *in vivo* experiments n = 10 of WT, n = 10 of Alport mice non-injected and n = 10 Alport mice injected with AFSC were used. Mice were injected with 1 × 10^6^ AFSC through the left ventricle at 3 months of age, before an onset of proteinuria as published^12^. Mice were sacrificed at 2 and 7 weeks post injections. In addition, Alport mice were injected with the equivalent number of EV (2.0 × 10^11^) as produced by 1 × 10^6^ AFSC (n = 8). Non-injected, aged matched Alport mice (n = 8) and WT mice (n = 5) served as control. 5 WT-Tek^*tdT*^ were used for flow cytometry data.

For cell tracking, 6 Alport mice were injected with AFSC pre-labeled with Qdot (or CM-DiI) immediately before the procedure (Invitrogen). The animals were killed at 5 days (n = 3), and 10 weeks (n = 3) after treatment, and the heart, kidney, liver, and lung were processed for FACS analysis as previously described^12^. A non-injected littermate served as the negative control throughout the analysis for each time point.

Renal function was assessed as previously described^12^. Of note, we refer to early stage AS when the level of proteinuria is <3 g/g (up to 3 months of age), to middle stage AS when proteinuria is ~10-13 g/g (around 4 months of age) and to late stage AS when proteinuria is >20 g/g (5 months of age and beyond). Experiments were performed in adherence to the NIH Guidelines for the Care and Use of Laboratory Animals and with Children’s Hospital Los Angeles Institutional Animal Care and Use Committee (IACUC) approval. Total number of mice used in experiments is 177: WT mice (n = 57), Alport mice (n = 90), WT-Tek^*tdT*^ (n = 13) Alport-Tek^*tdT*^(n = 23).

### Glomeruli and GEC isolation

Glomerulus isolation was performed as previously described^12^ and GEC from WT-Tek^*tdT*^ (n = 7) and Alport-Tek^*tdT*^ (n = 20) were isolated by further digesting the glomeruli with 0.25% trypsin (Gibco, ThermoFisher Scientific)/ 0.6% collagenase IV (Worthington) solution in media supplemented with phosphatase inhibitors (ThermoFischer Scientific) for 20′ at 37 °C. Cells were then passed through a 100μm strainer and GEC were flow sorted using FACSAria III (Becton Dickinson).

### EVs isolation, characterization and labeling

EVs were isolated from supernatants of AFSC cultured overnight in RPMI-1640 (Lonza) without serum^33^. Supernatants were centrifuged at 6,000 g for 20′ and ultracentrifuged at 100,000 g (Optima L-100K ultracentrifuge; Beckman Coulter) for 2 hours at 4 °C; pellets were resuspended in serum-free RPMI-1640 containing 1% DMSO and stored at −80 °C until use. EVs were characterized by cytofluorimetric analysis^38^, using fluorescein isothiocyanate (FITC), phycoerythrin (PE) or allophycocyanin (APC) conjugated rat antibodies. AFSC-EVs (1 × 10^9^ particles) were incubated for 15′ at 4 °C and immediately acquired by FACS analysis using a Guava easyCyte™ Flow Cytometer (Millipore) and analyzed with InCyte software (See list in Supplementary Material and Methods). Fluorochrome conjugated rat non-immune isotypic immunoglobulin G (Miltenyi Biotec) was used as a control. The size and distribution of the AFSC-EVs were analyzed using a NanoSight LM10 instrument (NanoSight Ltd.) equipped with the nanoparticle tracking analysis (NTA) 2.0 analytic software.

RNA isolated using the mirVana RNA isolation kit (Ambion), was analyzed using miScript Reverse Transcription Kit and miScript SYBR Green PCR Kit (both from Qiagen). Fold change in miRNA expression was calculated as 2^−∆Ct^ using the snoRNA RNU6b as normalizer^38^. A quantitative real-time polymerase chain reaction (qRT-PCR) was performed using a 96-well StepOne™ Real-Time System (Applied Biosystems) in order to analyze the EVs miRNA content. The sequence-specific oligonucleotide primers were all obtained from MWG-Biotech (www.mwg-biotech.com, see list in Supplementary Materials and Methods).

### Western Blotting, immunostaining, TEM, GEC quantification, VEGF and sVEGFR1 ELISA, PCR, PCR array, flow cytometry

Western blotting, immunostaining, PCR and PCR array, flow cytometry were performed as previously published^12,39^. Details are described in the Supplementary Materials and Methods. TEM analysis was performed as published^12^ and expression for VEGF and sVEGFR1 in glomerular extracts, serum and supernatants were assessed by ELISA according to manufacturers’ instructions (RayBiotech, #ELM-VEGF and #ELM-VEGFR1). GEC fenestration size was evaluated in 20 randomly selected TEM images per sample (n = 3) with a field of view at 28,000x. The surface areas of the fenestrations were quantified by ImageJ software (NIH). All measurements were done in a double-blinded fashion.

Alport (n = 3) and WT (n = 3) mice were administered with FITC-WGA lectin (from Triticum vulgaris; 6.25 mg/kg body wt; Ab20528; Abcam, MA) via an intracardiac injection and sacrificed after 30 minutes. Kidneys were snap frozen and 5μm sections were processed for confocal microscopy. Images were taken with Leica Zeiss 710 microscope and analyzed using the ZEN10 software.

### Co-immunoprecipitation assay

To evaluate VEGF/VEGFR1 interactions between VEGFR1 expressed by EVs and the excess of VEGF described in the *in-vitro* experiments, immunoprecipitation for VEGFR1 using VEGFR1 antibody and Protein G-agarose conjugate (Santa Cruz) was applied overnight at 4 °C. Immunoprecipitates were collected by centrifugation at 1,000 × g for 5′ at 4 °C, washed with PBS and resuspended in electrophoresis sample buffer, denatured and ran under reducing conditions as previously described^12^. The immunoblots were then probed for VEGF detection using VeriBlot-HRP conjugated secondary antibody (Abcam) and standard Western Blotting techniques as previously described^12^.

### *In vitro* co-culture of GEC-AFSC and GEC-EVs

GEC were seeded at 1 × 10^4^ cells/cm^2^ at 37 **°**C and 5% CO_2_ in medium supplemented with 0.1% VEGF, 0.1% ECGS, 0.1% Heparin, 0.1% EGF, 0.1% Hydrocortisone, 1% L-Glutamine, 1% antibiotic-antimycotic solution, 5% FBS (CellBiologics). GEC were overstimulated with recombinant VEGF (Gibco, ThermoFisher Scientific) at 100ng/ml. Simultaneously, AFSC (ration 1:1, on transwell inserts, Corning) or EVs and EV^*Flt1*−/−^ (10,000:1 ratio, on culture media) were co-cultured with GEC under the same growth conditions. Experiments were terminated at 24 hours and culture media and cells were collected for analysis. Integration of EVs into GEC was performed by applying EVs with green and red fluorescence tags derived from AFSC^*GFP*^ co-labeled with CM-DiI as previously reported^12^ to GEC overnight. Integration of EV into GEC was assessed by inverted fluorescence microscope (Leica DMI6000 B). Experiments were repeated in triplicate.

### Statistical analysis

All test populations were assumed to have Gaussian distribution and equal variance. Data shown in bar graphs are expressed as means ± SEM. Two-tailed Student’s *t*-test was used for comparisons between two groups. One-way ANOVA with Tukey’s *post hoc* test was applied for comparison of three or more groups for the same time point. Two-way ANOVA with Tukey’s *post hoc* test was applied for comparison of three or more groups between different time points (Fig. 4I,J). All statistical analysis was done with graphPad Prism 7.0a (GraphPad Software, Inc.). A p-value less than 0.05 was considered statistically significant.

### Data availability statement

All data generated or analyzed during this study are included in this published article (and its Supplementary Information files).

## Electronic supplementary material

Supplementary Data
