# Supplementary material for: Amniotic fluid stem cell-derived vesicles protect from VEGF-induced endothelial damage
Source: Sci Rep. 2017 Dec 4;7:16875. doi: 10.1038/s41598-017-17061-2 (PMC5715019; doi:10.1038/s41598-017-17061-2)

Title: Amniotic fluid stem cell-derived vesicles protect from VEGF-induced endothelial damage

S. Sedrakyan, PhD[1], V. Villani, PhD[1], S. Da Sacco, PhD[1], N. Tripuraneni, MS[1], S. Porta, MS[2], A. Achena, MD[1], M. Lavarreda-Pearce, BS[1], A. Petrosyan, PhD[1], H. Soloyan, MD[1], RE De Filippo, MD[1], B. Bussolati, MD PhD[2]*, L. Perin, PhD[1]*

1. GOFARR Laboratory for Organ Regenerative Research and Cell Therapeutics in Urology, Children's Hospital Los Angeles, Division of Urology, Saban Research Institute, University of Southern California, Los Angeles, California.
2. Department of Molecular Biotechnology and Health Sciences, University of Torino, Italy.

Supplementary Materials and Methods

Western Blot

Total protein from glomeruli and cultured GEC were collected and stored at −80°C in a radioimmunoprecipitation assay buffer supplemented with protease and phosphatase inhibitors (ThermoFischer Scientific) until use. Protein electrophoresis was performed on 4-20% Tris-Glycine gels and transferred onto a polyvinylidene fluoride 0.45-μm membrane (Millipore) and probed with antibodies with overnight incubation at 4°C. HRP-conjugated secondary anti-rabbit antibodies (Sigma-Aldrich) were applied it 1:20,000 ratio. Antigens were detected using the ECL Western Blotting detection reagents (Amersham Biosciences/GE Healthcare), impressed on Biomax Light Film (GE Healthcare). Data from 3 independent experiments were quantified by densitometry (all measurements were normalized against their corresponding housekeeping gene, β-actin).

Flow Cytometry

The expression and co-localization of VE-Cadherin, WT1 and PDGFRB markers with tdTomato was analyzed by flow cytometry. Flow cytometry analysis was performed in isolated glomerular cells from WT-Tek^{tdT} (n=5) using a BD FacsCanto. Data acquisition and analysis of all samples by flow cytometry were performed with BD FACSDiva 5.0.1 flow cytometry system and BD FACSDiva5.1.3 software.

Immunohistochemistry

Thin deparaffinized kidney sections (5 μm) were blocked in 3% BSA and immunostained for fluorescence microscopy with antibodies applied overnight at 4°C (see Table below). Alexa Fluor conjugated secondary antibodies (ThermoFisher Scientific) were applied at 1:500 dilution with 30' incubation at room temperature. A Leica DM RA fluorescent microscope was used in conjunction with Open Lab 3.1.5 software to image the staining.

Real-Time PCR and PCR Array

Total RNA from GEC sorted out of the Tek^{tdT} Alport and WT kidneys was extracted using Qiagen RNeasy kit according to the manufacturers' instructions. To characterize these GEC quantitative real-time PCR for AKT, CD31, PDGFRβ, VEGFR2 and WT1 was carried out using a Roche Light Cycler 480 and Light Cycler TaqMan Master Mix as previously described[12]. To evaluate GEC damage in vitro as well as the ability of AFSC to modulate VEGF gene expression in GEC was analyzed using the mouse endothelial cell biology RT^2 Profiler PCR Array System according to the manufacturers protocols (Qiagen) and as previously described[12]. Briefly, 250ng of total GEC RNA were used in the synthesis of template DNA using the RT^2 First Strand Kit according to the manufacturer's protocols (Qiagen). RT-PCR data were analyzed using the online real-time PCR analysis module by Qiagen.

Supplementary Figures

Supplementary Figure 1: Podocytes and VEGF expression within the glomerulus

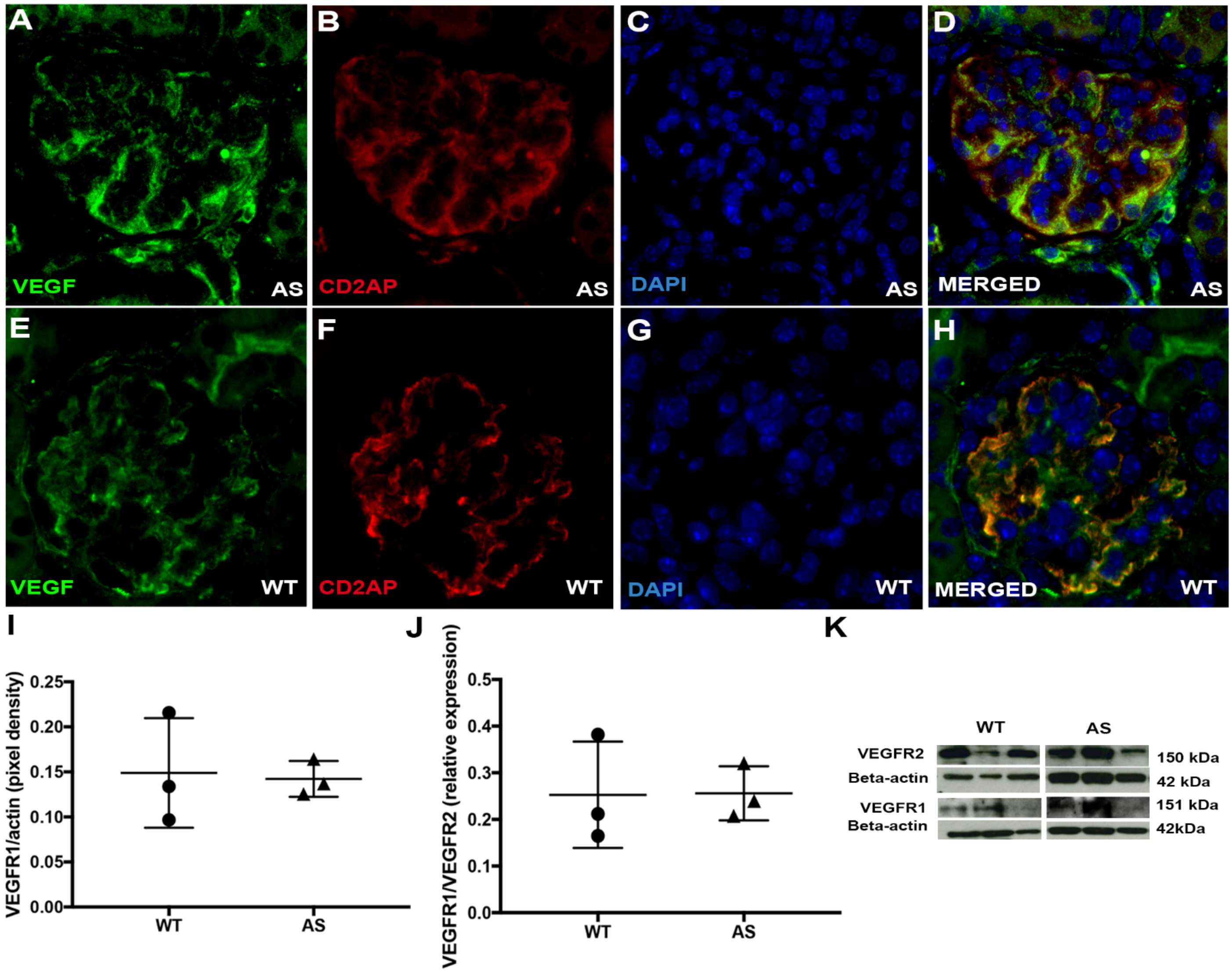

Representative images of VEGF expression (green; **A,E**) as produced by podocytes (CD2AP, red; **B,F**) are shown by co-immunostaining (yellow) within AS (**D**) and WT (**H**) glomeruli. Nuclear staining with DAPI (**C,G**, blue), image magnification (40x).

Graphs representing immunoblot data for VEGFR1 expression (**I**) and VEGFR1/VEGFR2 ratio (**J**) at 6 months of age (n=3 AS mice) as compared to their time-matched WT (n=3 WT mice) controls. No significant changes in the levels of VEGFR1 and VEGFR1/VEGFR2 ratio were detected. Immunoblots were quantified by densitometry (**K**). All VEGFR1 and VEGFR2 measurements were normalized against corresponding

housekeeping gene, β-actin, 42kDa. Two-tail student *t*-test was used to determine differences between WT and AS mice and scatter plot values are presented as mean ± SD (*p < 0.05).

Supplementary Figure 2. Characterization of GEC from WT-Tek^{tdT} mice

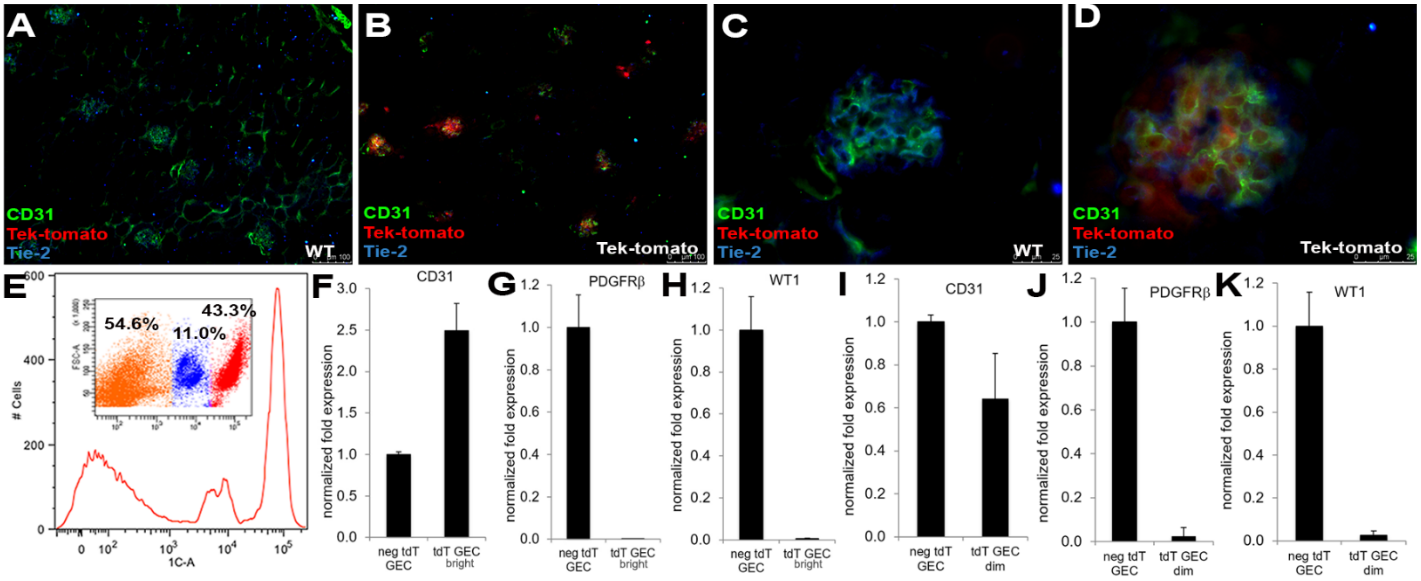

Representative images showing co-localization of *tdT* signal (red) with CD31 (green) and Tie-2 (blue) in glomeruli of WT-Tek^{tdT} mice (**A-D**). Expressions of CD31 and Tie-2 and *tdT* are predominantly co-localized within the glomeruli of Tek^{tdT} mice (**B,** 10x**; D,** 40x) while no co-expression is present in WT mice (**A,** 10x**; C,** 40x). **E.** Representative histogram of the *tdT* signal analyzed by flow cytometry showing the presence of two distinct *tdT* positive cell populations and their percent distributions (neg-*tdT* cells:54.6%, dim *tdT:*11.0% and bright *tdT:*43.3%). Higher mRNA expression for CD31 (**F**) was evident in bright *tdT*-GEC (isolated from n=3 mice), while neg-*tdT*-GEC (isolated from n=3 mice) expressed higher PDGFRβ (**G**) and WT1 (**H**). mRNA expression for CD31 (**I**), PDGFRβ (**J**), and WT1 (**K**) in the dim *tdT* cells (isolated from n=3 mice) against the neg-*tdT* cells (isolated from n=3 mice) shows lack of strong expression of endothelial marker CD31 and absence of PDGFRβ and WT1 in the dim *tdT* cells. Values are presented as mean ± SEM (*p < 0.05).

Supplementary Figure 3. Expression of glomerular markers in cells isolated from WT-Tek^{tdT} mice.

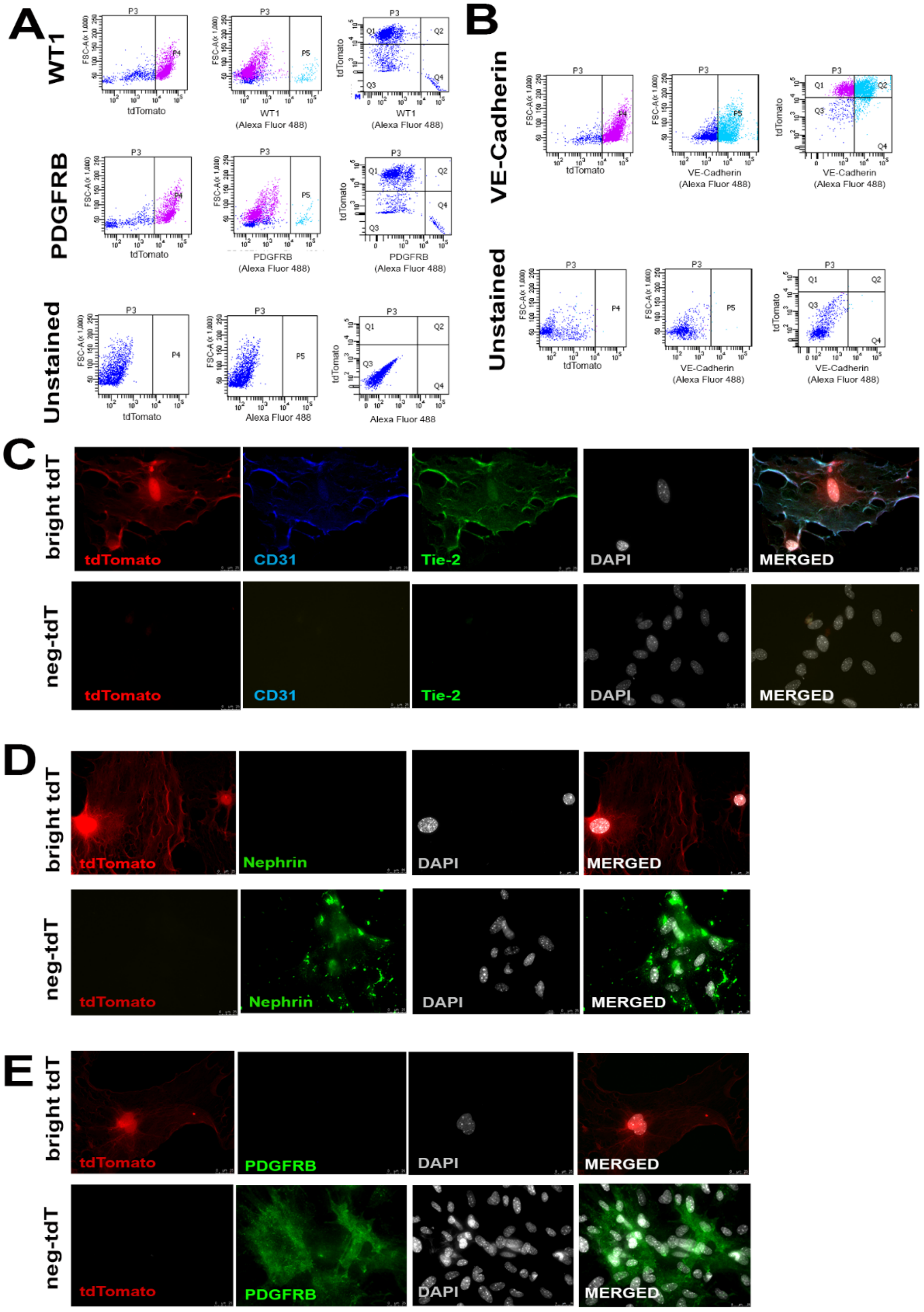

Flow cytometry showing lack of expression of PDGFRβ and WT1 in bright *tdT* cells (**A**) and co-expression of VE-Cadherin with *tdT* signal in bright *tdT* cells (**B**) confirming endothelial phenotype of bright *tdT* cells. Unstained plots are provided to show gating strategy. **(C)** Representative images of co-immunostaining for CD31 (bue) and Tie-2 (green) expression in bright *tdT* cells (red, upper panel) compared to neg-*tdT* cells

(lower panel); co-expression of these markers were evident in bright *tdT* cells and absent in neg-*tdT* cells. (**D**) Representative images of co-immunostaining for Nephrin (green) expression in bright *tdT* cells (red, upper panel) compared to neg-*tdT* cells (lower panel); co-expression of these markers were absent in bright *tdT* cells and evident in neg-*tdT* cells. (**E**) Representative images of co-immunostaining for PDGFRβ (green) expression in bright *tdT* cells (red, upper panel) compared to neg-*tdT* cells (lower panel); co-expression of these markers were absent in bright *tdT* cells and evident in neg-*tdT* cells. Nuclear staining with DAPI (grey), image magnification (40x).

Supplementary Figure 4. AFSC homing

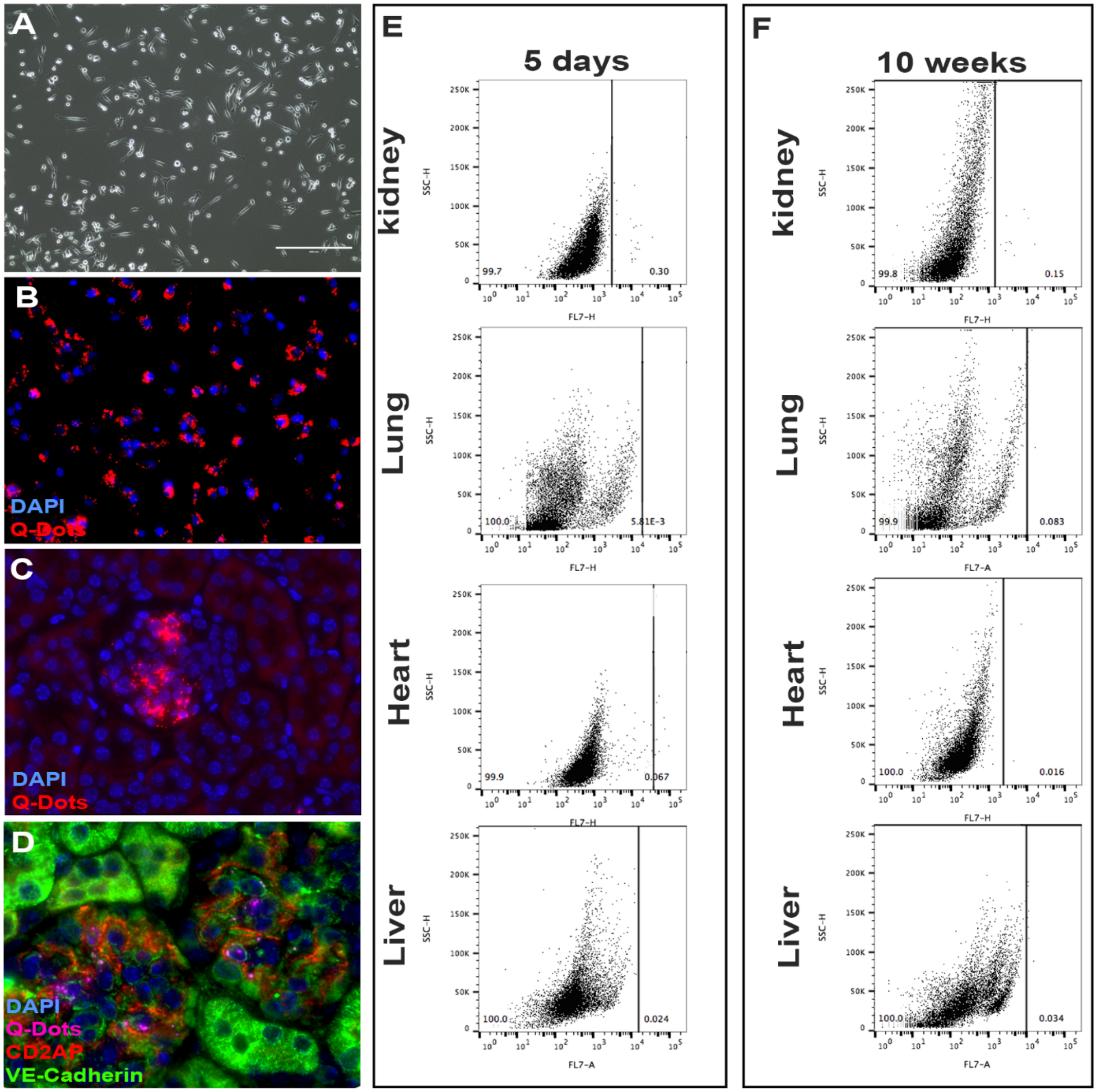

Representative bright field image of AFSC in culture before being tagged with a fluorescent dye (Q-dot) (**A,** 10x) and after tagging for in vivo detection (**B,** 10x). AFSC (red) homed predominantly within glomeruli of AS mice, detected by fluorescence microscopy at 5 days (**C-D**) and by FACS (5 days and 10 weeks, **E-F**) post injection. AFSC (magenta) were found localized in close association with the endothelial cells (VE-Cadherin, green) as opposed to podocytes (CD2AP, red) within the glomerulus (**D**, 40X). Nuclear staining with DAPI (blue). Representative scatter graphs from flowcytometric analysis of the distribution of the Qdot positive AFSC within the different organs at 5 days (n=3) (**E**) and 10 weeks (n=3) (**F**) post injection.

Supplementary Figure 5. AFSC rescue GEC damage induced by VEGF overexpression

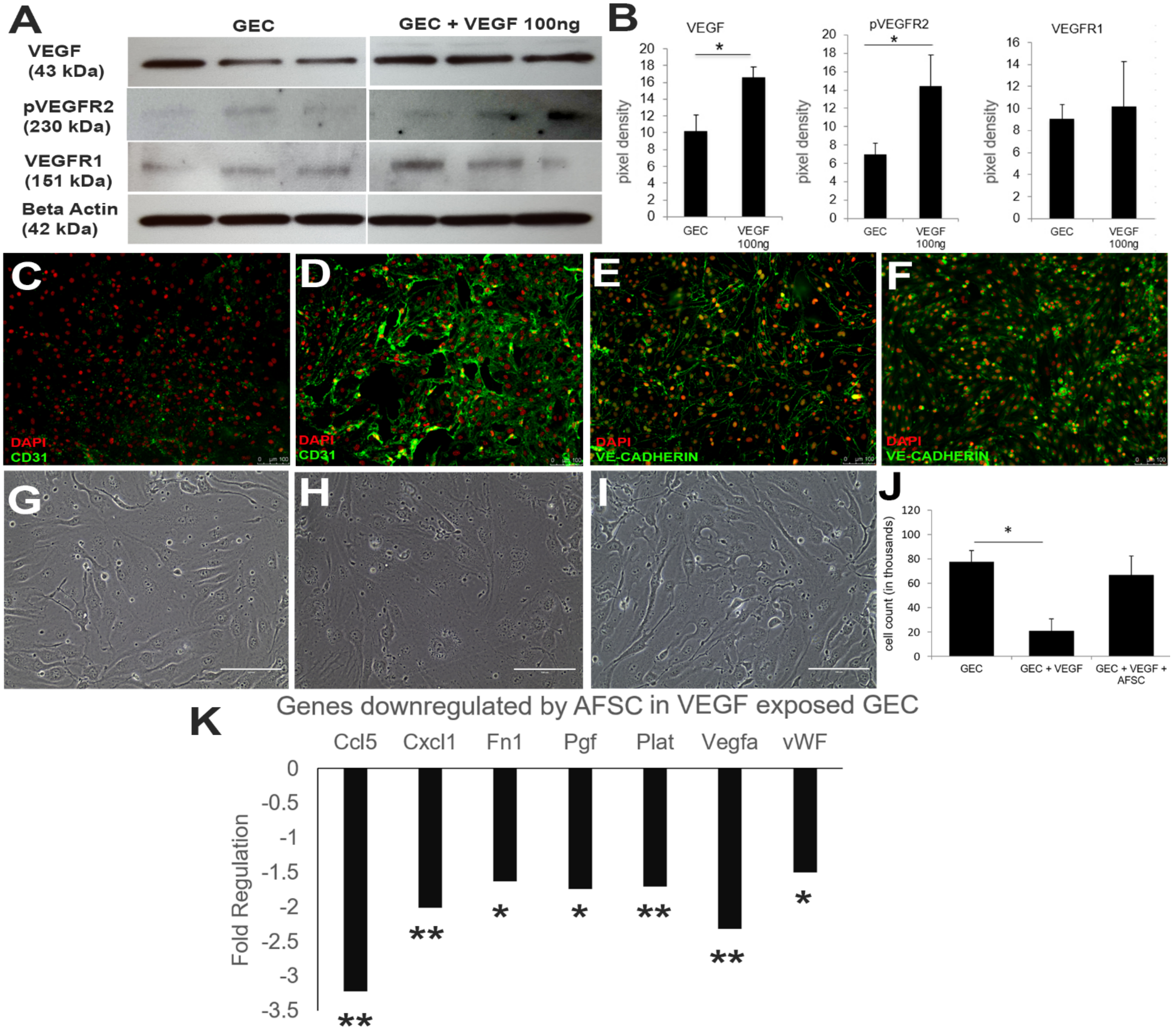

GEC ($2.0x10^4$cell/cm^2) were exposed to 100ng/ml recombinant VEGF for 24hrs to simulate peak VEGF conditions observed in Alport mice at 3 months of age. GEC damage and disruption of VEGF signaling and effects of AFSC were assessed by immunoblotting and immunohistochemistry (**A-F**). VEGF overstimulation caused increased expression of VEGF (43KDa, dimer) and pVEGFR2 (230KDa), whereas VEGFR1 (151KDa) remained unchanged (**A-B**). Immunoblot measurements were normalized against their corresponding housekeeping gene, β-actin, 42KDa. Representative immunohistochemistry images showing increased CD31 staining (**C-D,** 10x, green) and disrupted pattern for VE-Cadherin (**E-F,** 10x, green) in GEC exposed to VEGF as compared with non-stimulated controls. Nuclear staining with DAPI (red). Representative bright field images of GEC (**G,** 10x), GEC exposed to VEGF (**H,** 10x) and after treatment with AFSC (**I**, 10x). These observations showed disrupted GEC morphology with VEGF overstimulation (**H**) compared to control non-stimulated GEC (**G**). GEC co-cultured with AFSC (at 1:1 ratio on transwell) on the other hand improved cell morphology (**I**). **J.** Graph representing reduced GEC counts after 24hrs of VEGF overstimulation but were ameliorated with AFSC treatment. **K.** Endothelial Biology RT^2 Profiler PCR-array (Qiagen, SA Biosciences) demonstrating the modulation of specific endothelial genes, including VEGF, by AFSC. PCR data were analyzed by RT^2 Profiler PCR Array Data Analysis version 3.5 by Qiagen. Experiments were repeated in triplicate and one-way ANOVA with Tukey's *post hoc* test was used to analyze the data between 3 or more groups (**J**); student *t*-test was used to compare the means between two groups. Values are presented as mean ± SEM (*$p < 0.05$).

Supplementary Figure 6. AFSC-EV miRNA characterization and AFSC-EV uptake by GEC

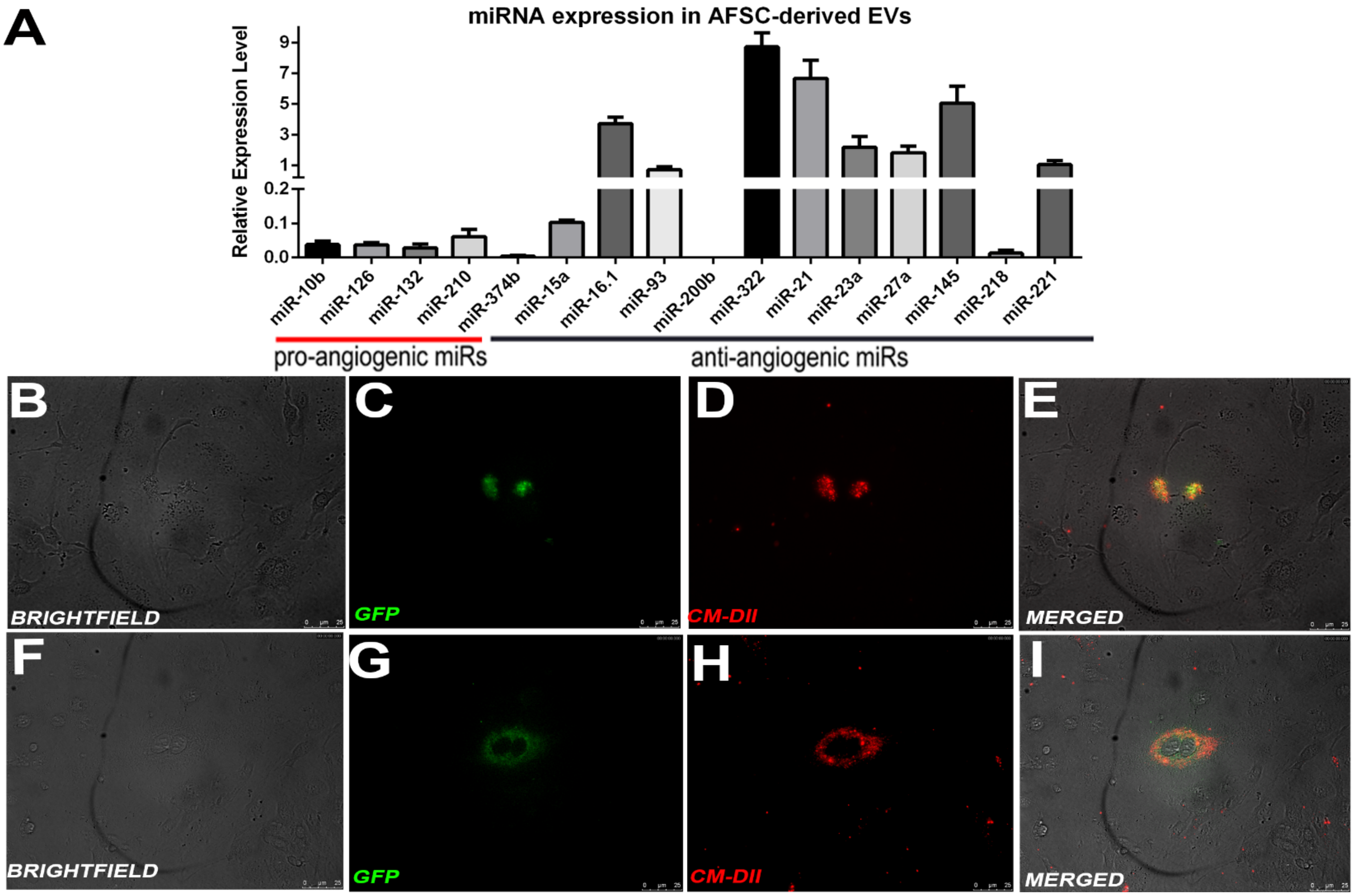

A. Fold change analysis of miRNAs expression in AFSC-derived EVs. The snoRNA RNU6b was used as normalizing reference control. Fold change in miRNA expression was calculated as $2^{-\Delta Ct}$ using the geometric mean in Ct values as normalizer. EVs are derived from the same cell line and the analysis was repeated in quadruplicate.

In order to demonstrate uptake of AFSC-derived EVs by GEC, GEC were incubated with AFSC-labeled-EVs for 18 hours. **B-I.** Representative images (bright field and fluorescence) of GEC in culture in the presence of GFP-EVs (green) derived from AFSC^{GFP} and also labeled with CM-DiI (red) before exposure, immediately after exposure (0hrs) and 18hrs later. As evident in the merged picture (**E**, 10x and **I,** 40x), double-labeled EVs are present within the perinuclear of GEC. Values are presented as mean ± SEM.

Table 1 - List of Antibodies for flow cytometry, Western Blotting and immunostaining (cells)

Antibody	Company	Catalogue Number	Dilution
PDGFRβ	Abcam	ab5511-100	Flow cytometry: 1:100
WT1	Santa Cruz	sc-192	Flow cytometry: 1:100
VEGF	Abcam	ab46154	WB: 1:100<br>IF: 1:200
VEGFR1	Abcam	ab2350	WB: 1:200
VEGFR2	Novus Biological	NB100-627SS	WB: 1:5000
pVEGFR2(Tyr951)	Cell signaling	4991	WB: 1:1000
Beta-actin	Gentex	109639	WB: 1:1000
VE-Cadherin	Abcam	ab33168	Flow cytometry: 1:50<br>IF: 1:100
CD31	Pharmingen	553370	IF: 1:200
CD2AP	Santa Cruz	34229	IF: 1:50
WGA	Abcam	Ab20528	in vivo injection: 6.25 mg/kg body wt
PV1	LS BioSciences	LS-C58132	IF: 1:50
Akt	Cell signaling	9272	WB: 1:1000
pAkt(Ser473)	Cell signaling	9271	WB: 1:1000
ERK1/2(p44/42 MAPK)	Cell signaling	4695	WB: 1:1000
pERK1/2(p44/42 MAPK) (Thr202/Try204)	Cell signaling	4377	WB: 1:1000

Table 2 - List of Antibodies for Flow Cytometry (EVs)

Antibody	Company	Catalogue Number	Dilution
CD29	Miltenyi Biotec	130-102-975	1:300
CD44	Miltenyi Biotec	130-102-977	1:300
CD73	Miltenyi Biotec	130-103-054	1:300
CD90	Miltenyi Biotec	130-102-636	1:300
CD105	Miltenyi Biotec	130-102-915	1:300
CD146	Miltenyi Biotec	130-102-846	1:300
CD9	Miltenyi Biotec	130-102-579	1:300
CD24	Miltenyi Biotec	130-103-370	1:300
TIE2	eBioscience	12-5987-81	1:300
CD63	R&D Systems	MAB5417	1:300
VEGFR1	R&D Systems	AF471	1:300
VEGFR2	R&D Systems	AF644	1:300
NEUROPILIN-I	R&D Systems	AF566	1:300
NEUROPILIN-II	R&D Systems	AF567	1:300

Table 3 – List of primers for qPCR (cells/tissue)

Primer	Sequence	Probe
AKT	5’- tcgtgtggcaggatgtgtat -3’<br>5’- acctggtgtcagtctcagagg-3’	45
CD31	5’-actcgacaggatggaaatcac-3’<br>5’-cggtgttcagcgagatcc-3’	45
PDGFRβ	5’-tcaagctgcaggtcaatgtc-3’<br>5’-ccattggcagggtgactc-3’	32
VEGF	5’-caggctgctgtaacgatgaa-3’<br>5’-gctttggtgaggtttgatcc-3’	9
VEGFR1	5’-atccctcggccaacaatc-3’<br>5’-cattctcagtgcagaagtcatacc-3’	21
VEGFR2	5’-cagtggtactggcagctagaag-3’<br>5’-acaagcatacgggcttgttt-3’	68
WT1	5’-cagatgaacctaggagctaccttaaa-3’<br>5’-tctgcccttctgtccatttc-3’	3
18S	5’-aaatcagttatggttcctttggtc-3’<br>5’-gctctagaattaccacagttatccaa-3’	55

Table 4 – List of primers for miRNAs (EVS)

miRNA	Sequence
miR-10b-5p	5-uacccuguagaaccgaauuugug-27
miR-126a-3p	46-ucguaccgugaguaauaaugcg-67
miR-132-3p	42-uaacagucuacagccauggucg-63
miR-210-3p	66-cugugcgugugacagcggcuga-87
miR-15a-5p	15-uagcagcacauaaugguuugug-36
miR-16-1-5p	16-uagcagcacguaaauauuggcg-37
miR-93-5p	15-caaagugcuguucgugcagguag-37
miR-200b-3p	45-uaauacugccugguaaugauga-66
miR-322-5p	23-cagcagcaauucauguuuugga-44
miR-21a-5p	18-uagcuuaucagacugauguuga-39
miR-23a-3p	46-aucacauugccagggauuucc-66
miR-27a-3p	56-uucacaguggcuaaguuccgc-76
miR-145a-5p	7-guccaguuuucccaggaaucccu-29
miR-218-1-5p	25-uugugcuugaucuaaccaugu-45
miR-221-3p	60-agcuacauugucugcuggguuuc-82

Supplementary Figure 7. Original blots for Main Manuscript Figure 1 and Figure 4

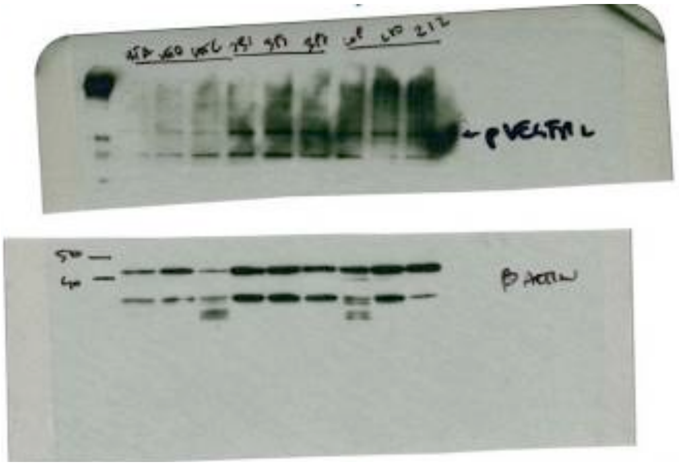

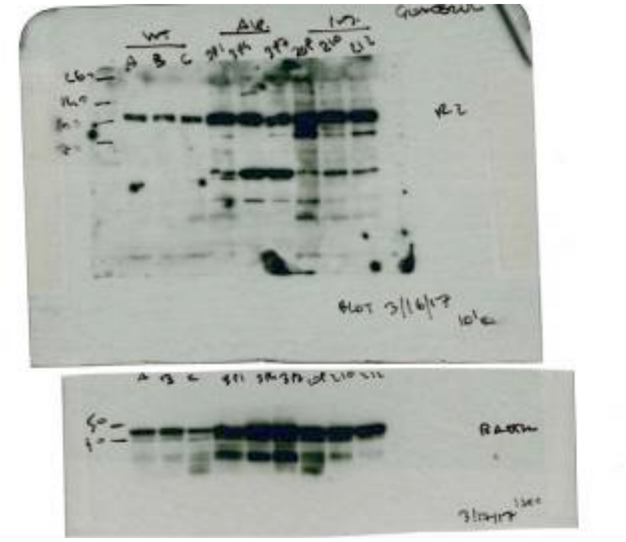

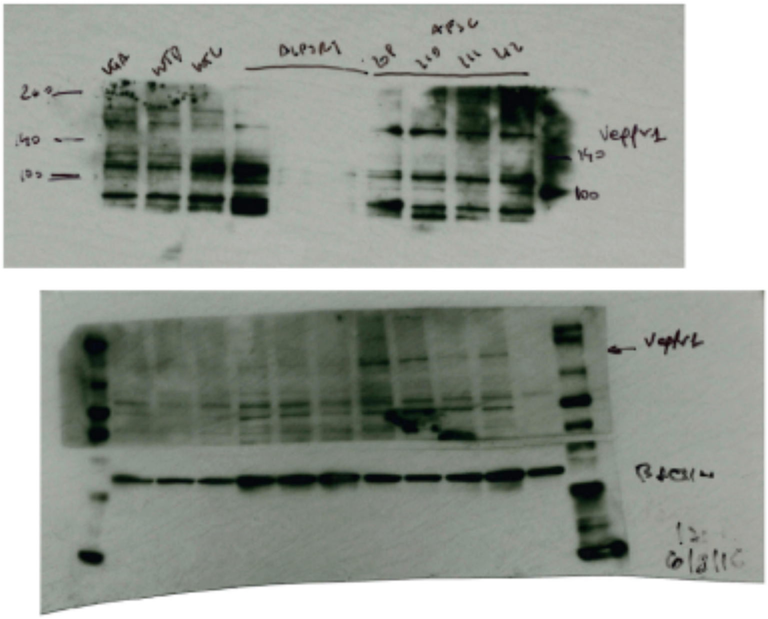

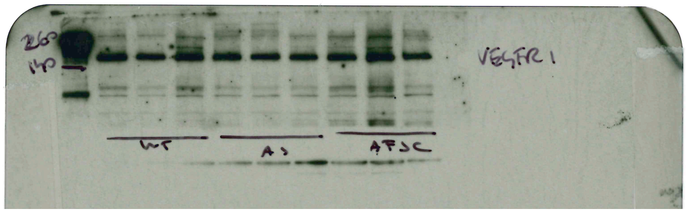

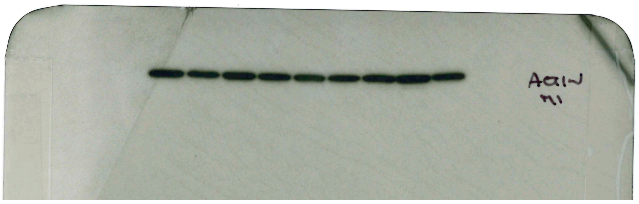

Supplementary Figure 8. Original blots for Main Manuscript Figure 3

Suppl. Figure 8-1a: immunoblot images for WT AKT, pAKT in Fig 3F

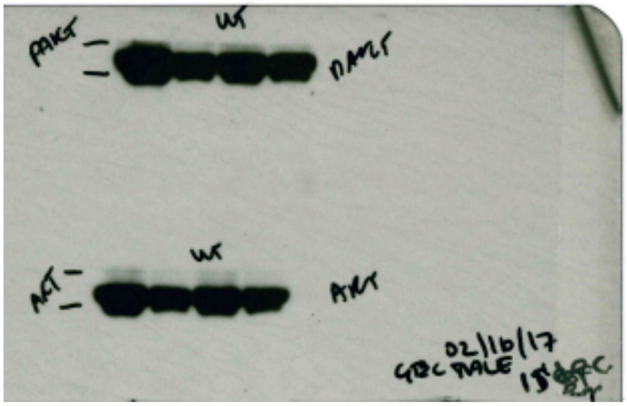

Suppl. Figure 8-1b: immunoblot images for AS 2M and 4M AKT, pAKT in Fig 3F exposure for 4M

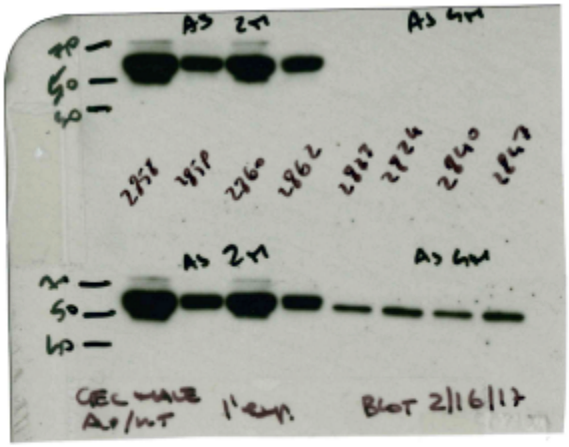

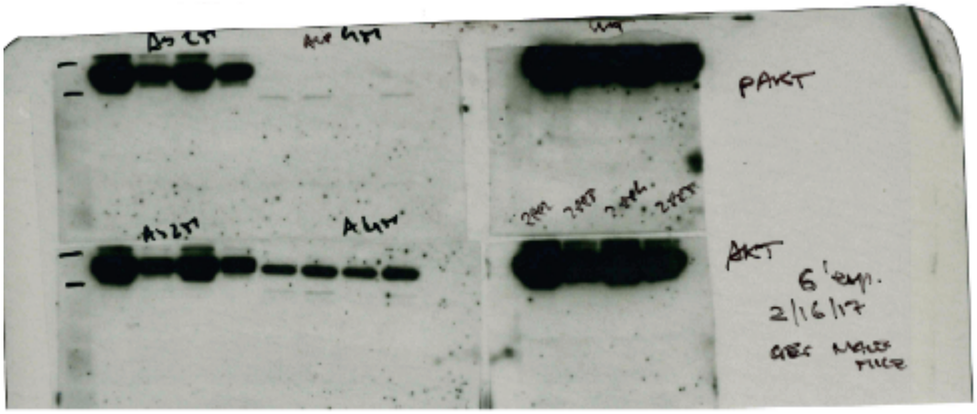

Longer exposure for pAKT

Suppl. Figure 8-1c: immunoblot images for AS 3M AKT, pAKT in Fig 3F

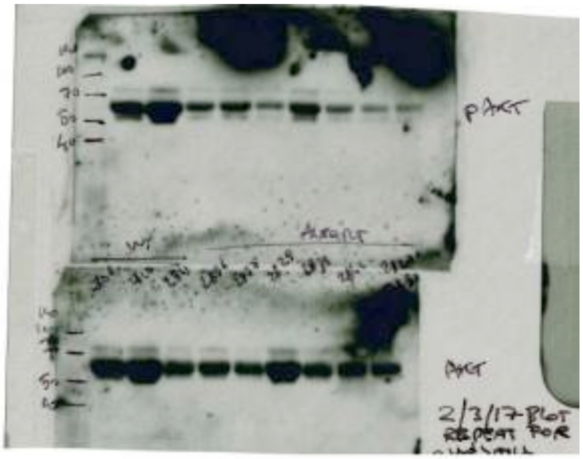

Suppl. Figure 8-2a: immunoblot images for WT ERK and pERK in Fig 3F

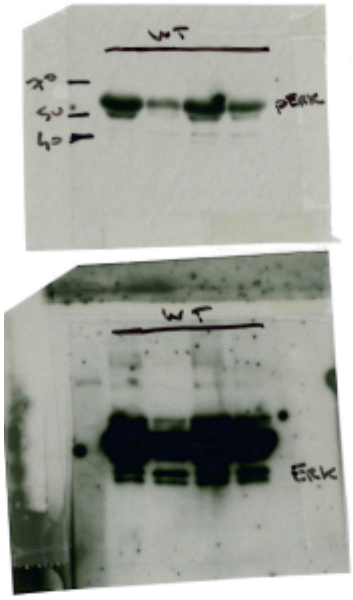

Suppl. Figure 8-2b: immunoblot images for AS 2M and 4M ERK and pERK in Fig 3F R1

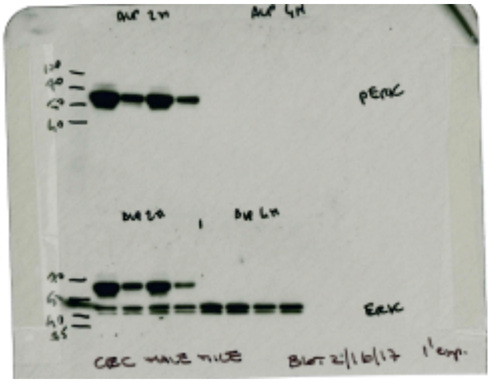

Suppl. Figure 8-2c: immunoblot images for AS 3M ERK and pERK in Fig 3F

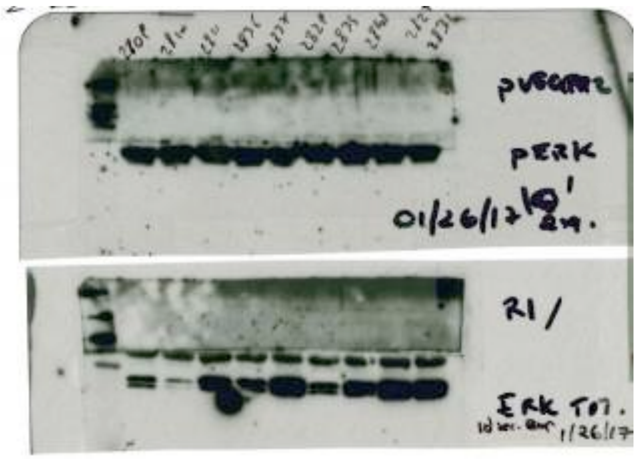

Suppl. Figure 8-3a: immunoblot images for VEGFR1 and *b*-actin for AS 2M, 4M in Fig 3F

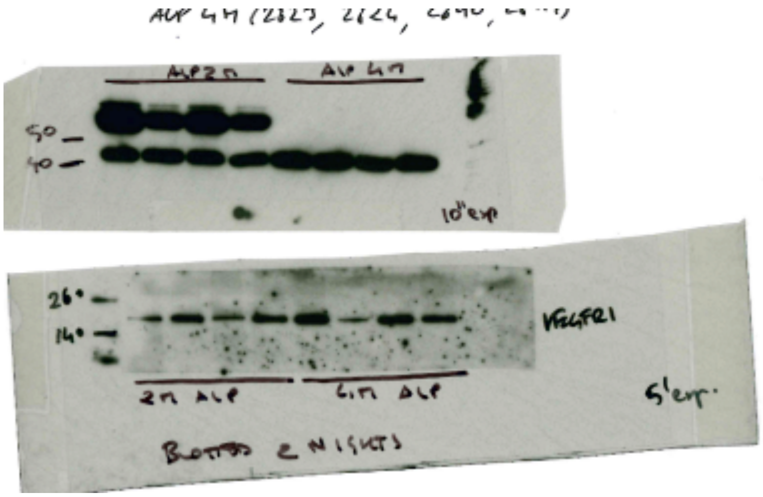

Suppl. Figure 8-3b: immunoblot images for VEGFR1 and *b*-actin for WT and AS 3M in Fig 3F

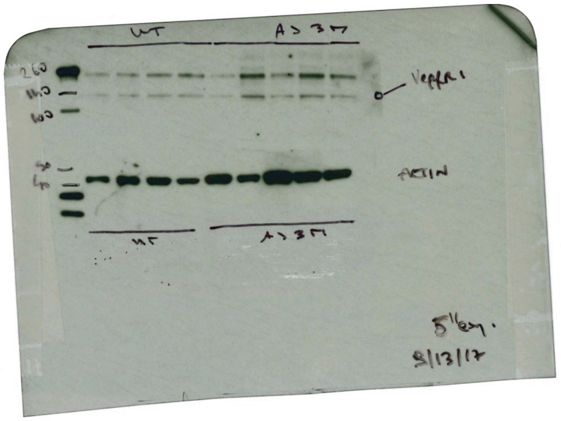

Supplementary Figure 9 – Original blots for Main Manuscript Figure 6

Suppl. Figure 9-1: immunoblot image for VEGFR1 in Fig 6C

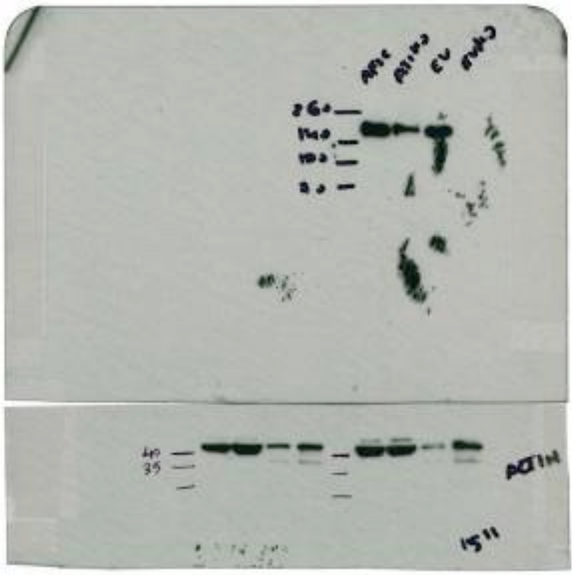

Suppl. Figure 9-2: immunoblot image for VEGFR1 in Fig 6D

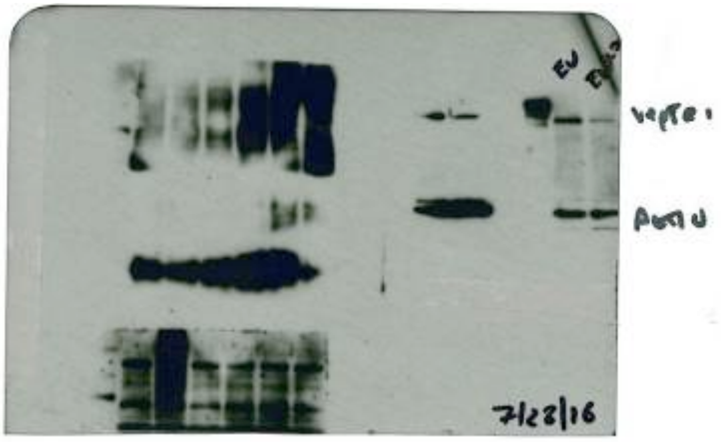

Suppl. Figure 9-3: immunoblot image for VEGFR2 in Fig 6M

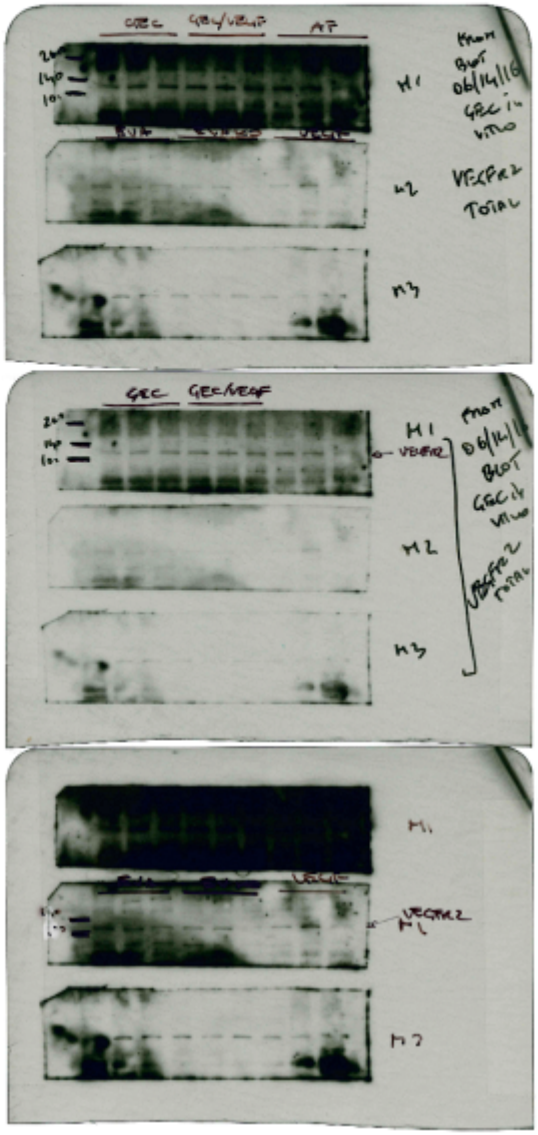

Suppl. Figure 9-4: immunoblot image for pVEGFR2 in Fig 6M

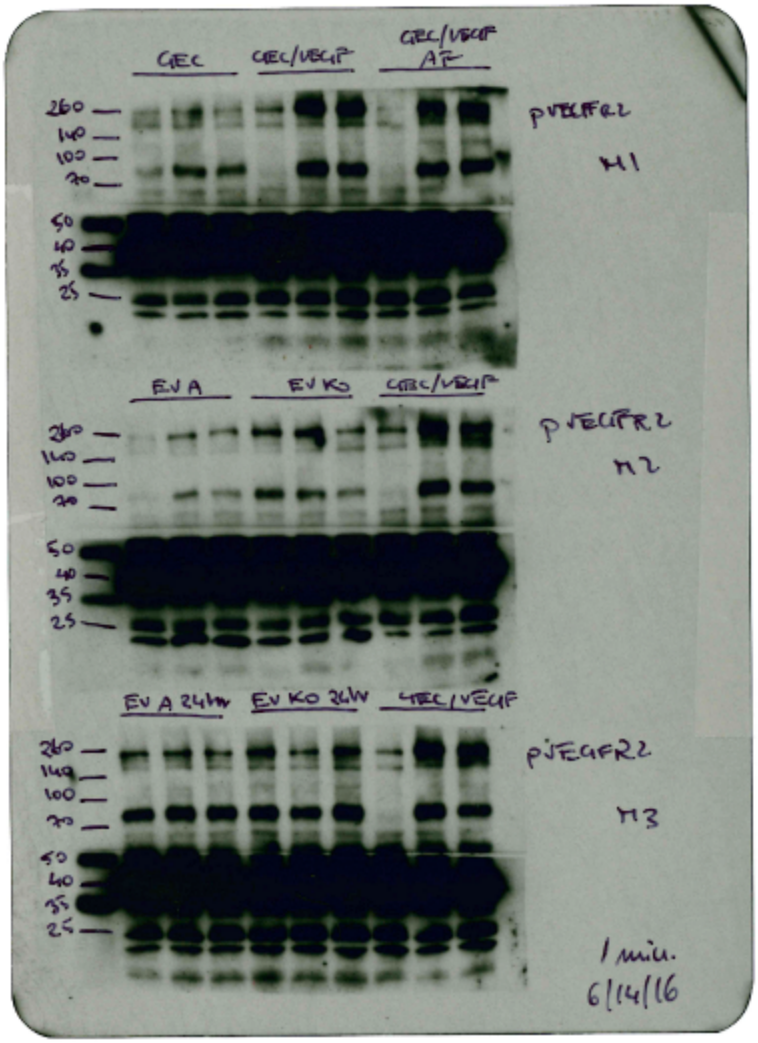

Suppl. Figure 9-5: immunoblot image for *b*-actin for VEGFR2 and pVEGFR2 in Fig 6M

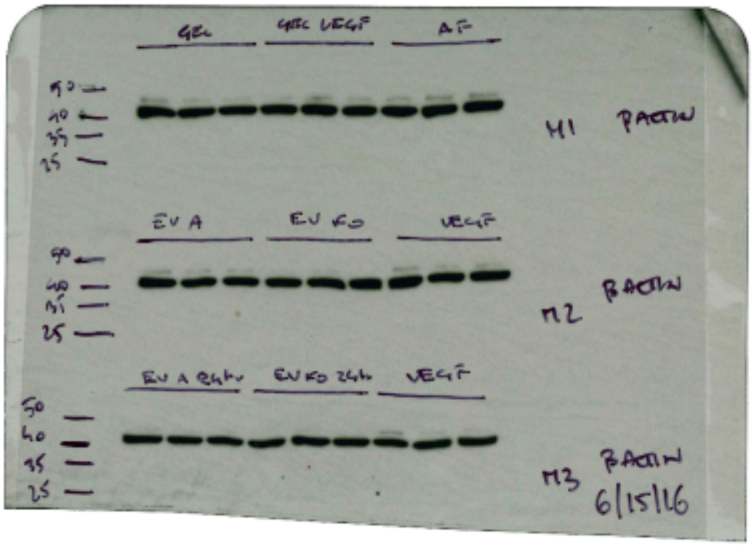

Suppl. Figure 9-6: immunoblot image for VEGFR1 and corresponding *b*-actin in Fig 6M

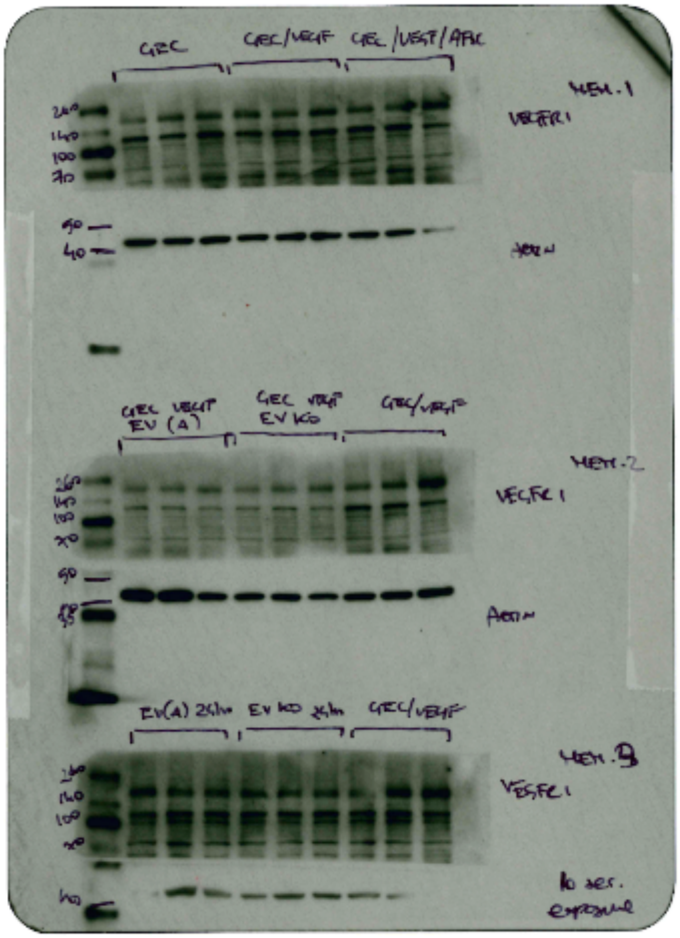

Suppl. Figure 9-7: immunoblot image for VEGFR2 and pVEGFR2 in Fig 6N

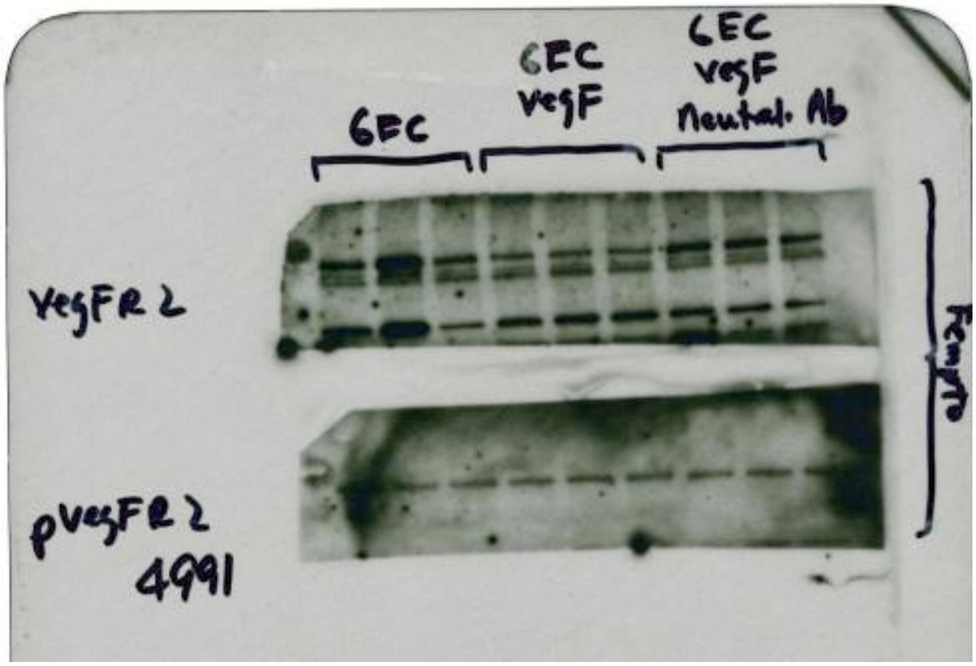

Suppl. Figure 9-8: immunoblot image for VEGFR1 and corresponding *b*-actin Fig 6N

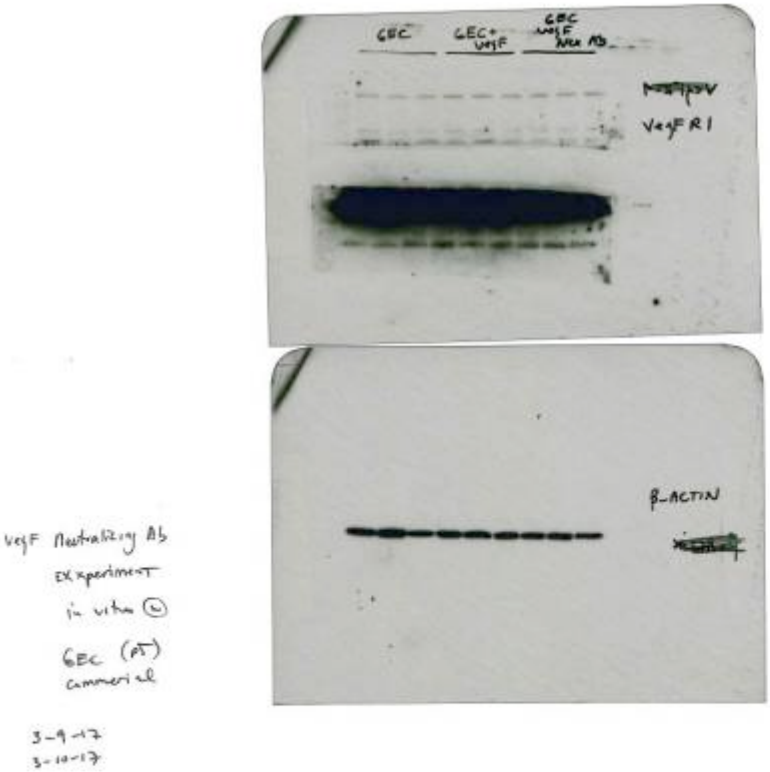

Supplementary Figure 10 – Original blots for Main Manuscript Figure 7

Suppl. Figure 10-1: immunoblot image for VEGF in Fig 7A

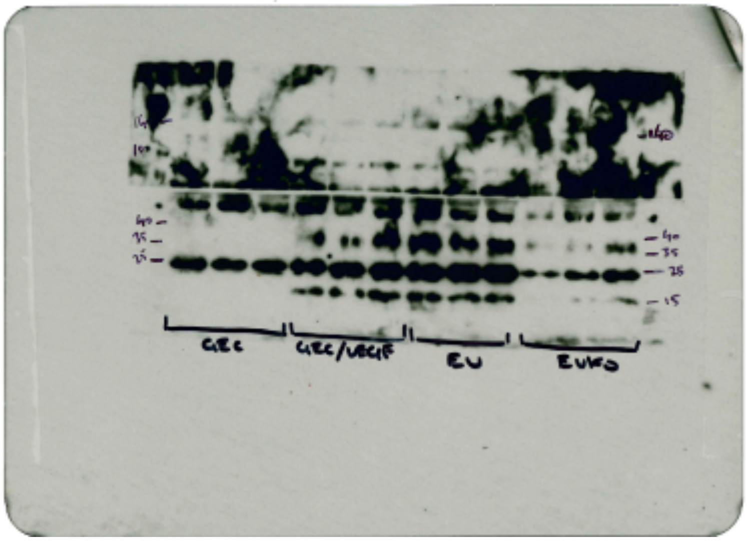

Suppl. Figure 10-2: immunoblot image for VEGF in Fig 7B

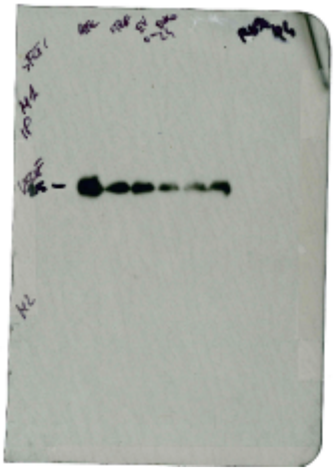

Suppl. Figure 10-3: immunoblot image for VEGF in Fig 7C

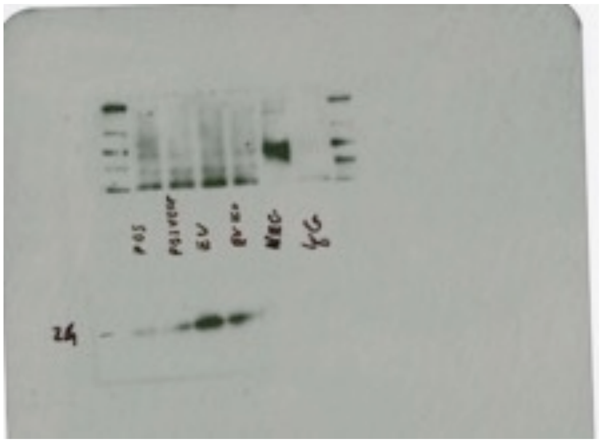

Supplementary Figure 11. Original blots for Main Manuscript Supplementary Figure 1

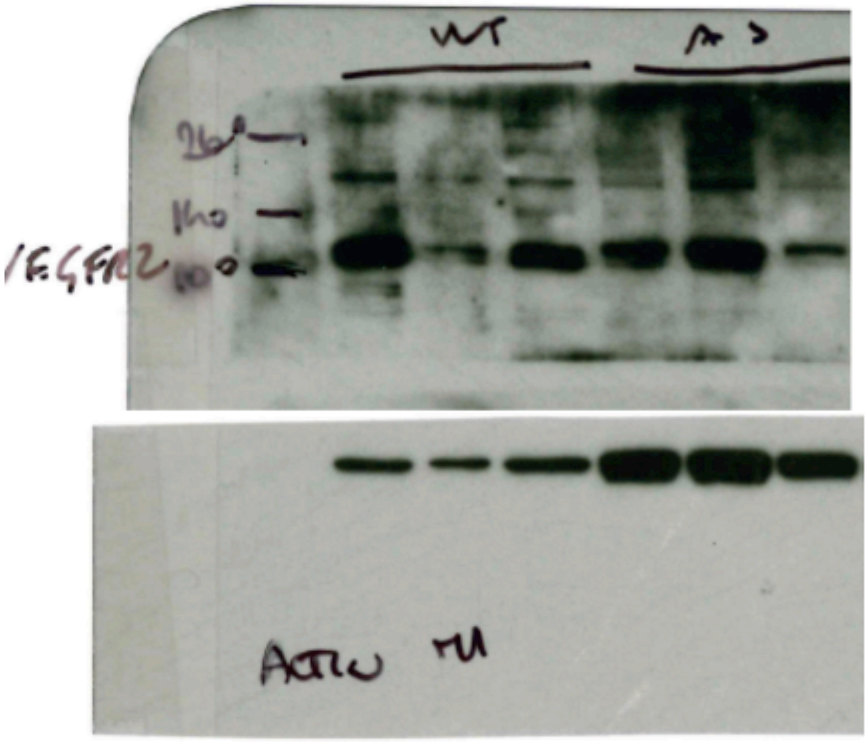

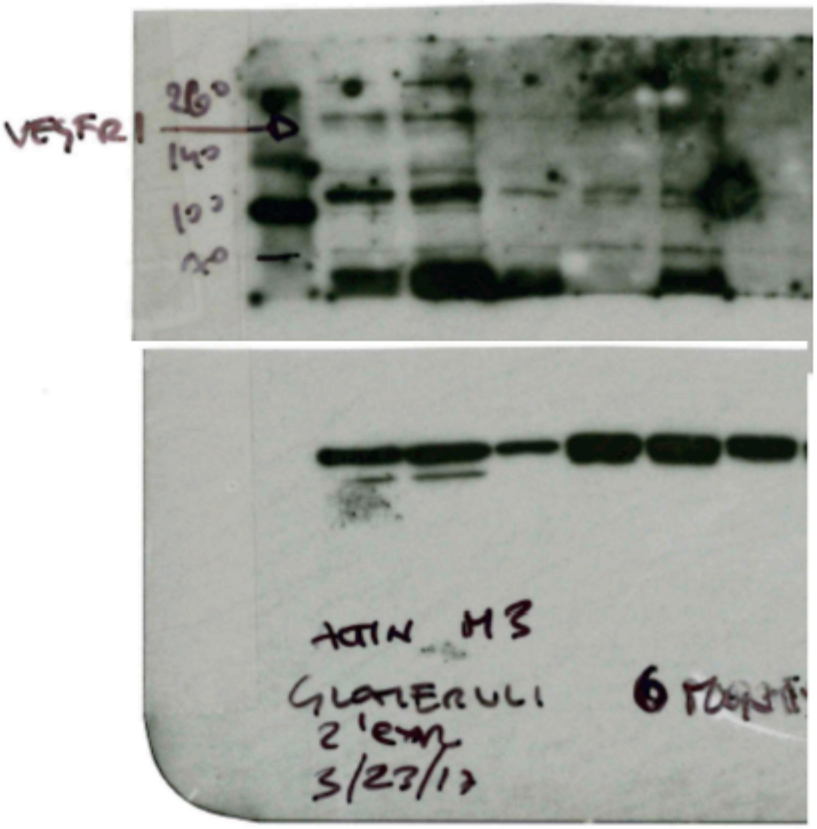

Supplementary Figure 12. Original blots for Main Manuscript Supplementary Figure 5

Suppl. Figure 12-1: immunoblot image for *b*-actin in Suppl. Fig 5A

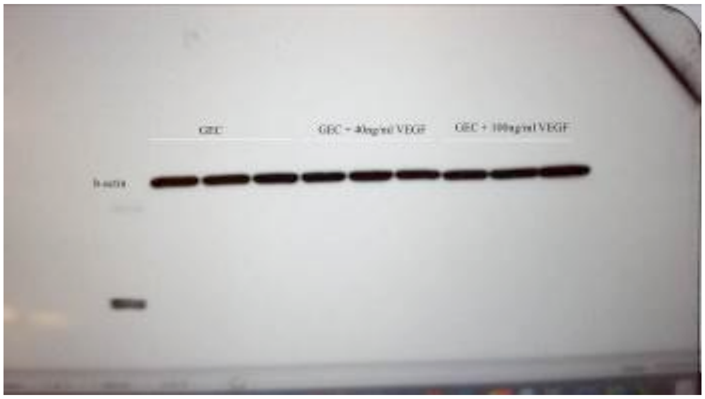

Suppl. Figure 12-2: immunoblot image for VEGF in Suppl. Fig 5A

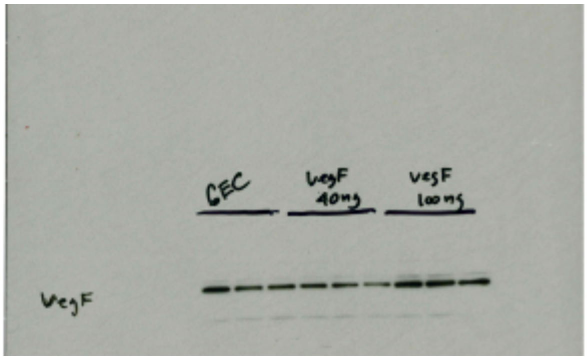

Suppl. Figure 12-3: immunoblot image for pVEGFR2 in Suppl. Fig 5A

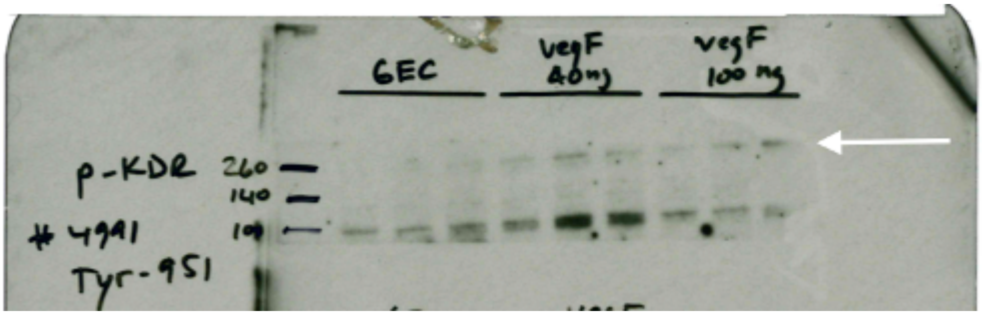

Suppl. Figure 12-4: immunoblot image for VEGFR1 in Suppl. Fig 5A

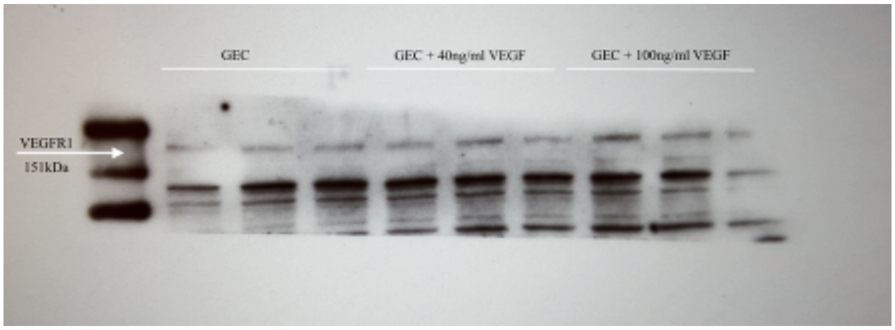

Supplement: Supplementary file 1 — Supplementary Data [file 41598_2017_17061_MOESM1_ESM.pdf]
